# Opposite forms of adaptation in mouse visual cortex are controlled by distinct inhibitory microcircuits

**DOI:** 10.1038/s41467-022-28635-8

**Published:** 2022-02-24

**Authors:** Tristan G. Heintz, Antonio J. Hinojosa, Sina E. Dominiak, Leon Lagnado

**Affiliations:** Sussex Neuroscience, School of Life Sciences, University of Sussex, Brighton, BN1 9QG UK

**Keywords:** Neuroscience, Sensory processing

## Abstract

Sensory processing in the cortex adapts to the history of stimulation but the mechanisms are not understood. Imaging the primary visual cortex of mice we find here that an increase in stimulus contrast is not followed by a simple decrease in gain of pyramidal cells; as many cells increase gain to improve detection of a subsequent decrease in contrast. Depressing and sensitizing forms of adaptation also occur in different types of interneurons (PV, SST and VIP) and the net effect within individual pyramidal cells reflects the balance of PV inputs, driving depression, and a subset of SST interneurons driving sensitization. Changes in internal state associated with locomotion increase gain across the population of pyramidal cells while maintaining the balance between these opposite forms of plasticity, consistent with activation of both VIP->SST and SST->PV disinhibitory pathways. These results reveal how different inhibitory microcircuits adjust the gain of pyramidal cells signalling changes in stimulus strength.

## Abstract (web summary)

The authors describe the role of inhibitory microcircuits in the visual cortex of mice in adaptation to contrast. They show how external stimuli and internal state interact to adjust processing in the visual cortex.

## Introduction

The sensory world is dynamic and so are the properties of the neural circuits that process the information it provides^1^. Adjustments in these circuits driven by the recent history of stimulation are generally termed adaptation and are thought to improve the efficiency with which information can be extracted in a changing sensory environment^2^. In the visual system, the most commonly observed form of adaptation is a decrease in sensitivity to a constant feature of the input. This feature might be the average luminance or contrast^3^ as well as statistical properties of higher-order, such as spatial patterns^4^. Adaptation to contrast as a *depressing* response has been observed throughout the visual system, beginning in the synaptic output of retinal bipolar cells^5^ through retinal ganglion cells^6^, the dorsal lateral geniculate nucleus^7,8^ and primary visual cortex, V1^3,7,9^. But adaptation in V1 is not simply inherited from the retina: local processing also controls time-dependent changes in the gain of pyramidal cells^10,11^ and increases in sensitivity of some neurons can occur simultaneously with decreases in the sensitivity of others^12,13^. A number of synaptic and network processes are likely to generate adaptive effects in V1 and here we investigate the roles of inhibitory microcircuits during adaptation to contrast.

Inhibition plays a key role in controlling the gain and dynamics of signals in sensory cortices but our understanding of the mechanisms is limited^7,14,15^. This is in part because most studies in vivo have used anaesthetized animals in which inhibitory synaptic transmission is weaker than in awake animals^11,16^. The use of awake animals has also revealed that the recent history of the stimulus is not the only variable controlling the gain of sensory responses; these are also strongly modulated by changes in the internal state of the animal, reflected in behaviours such as the sleep-wake cycle, locomotion and arousal^15,17^. In V1, for instance, locomotion increases the gain of pyramidal cells (PCs) through disinhibition pathways^18–21^. We therefore also need to understand how endogenous changes in cortical state interact with adaptation to the external stimulus.

To investigate how inhibitory circuits control the gain of responses in V1 we have used multiphoton imaging in mouse lines expressing Cre-drivers in each of the three broad classes of interneuron, expressing one of parvalbumin (PV), somatostatin (SST) or vasoactive intestinal polypeptide (VIP)^22^. We find that PCs experience opposite forms of plasticity: while one subset respond to an increase in contrast with high initial gain and then depress over periods of seconds, an equally numerous subset respond with low initial gain but then gradually sensitize so that the average activity across the population is relatively constant. Adaptation over a similar time-course occurs in interneurons but while VIP-positive and PV-positive cells sensitize, there are two subsets of SST-interneurons which exhibit opposite forms of plasticity. To understand the circuits underlying these varying dynamics, we used optogenetics to activate or inhibit the major classes of interneuron and these results lead us to propose that there are two levels at which adaptation in PCs is controlled. The first reflects the balance of *direct* inputs received from PV interneurons driving depression of PCs and SST interneurons driving sensitization of PCs. The second level of control reflects modulation of these direct inputs through two disinhibition pathways, VIP→SST and SST→PV. Locomotion increases the gain across the population of PCs while maintaining the balance between depressing and sensitizing forms of adaptation, so we propose that both these disinhibition pathways become engaged during locomotion.

## Results

### Opposite forms of adaptation across the population of pyramidal cells

To investigate contrast adaptation in V1 we used awake mice in which inhibitory activity was intact^16^. PCs expressing the calcium reporter GCaMP6f under the CaMKII promoter were imaged in Layer 2/3 and stimuli consisted of drifting sinusoidal gratings presented for 10 s (20° visual field, 80% contrast, 1 Hz, see “Methods” section). The duration of the stimulus was chosen based on the similar time-scale of adaptive effects observed in the retina^23,24^ and in V1 of awake mice^11^. The responsivity of neurons in V1 is strongly dependent on locomotion^19^ so we began by confining our analysis to measurements made while the mouse was running on a trackball.

**Fig. 1:**
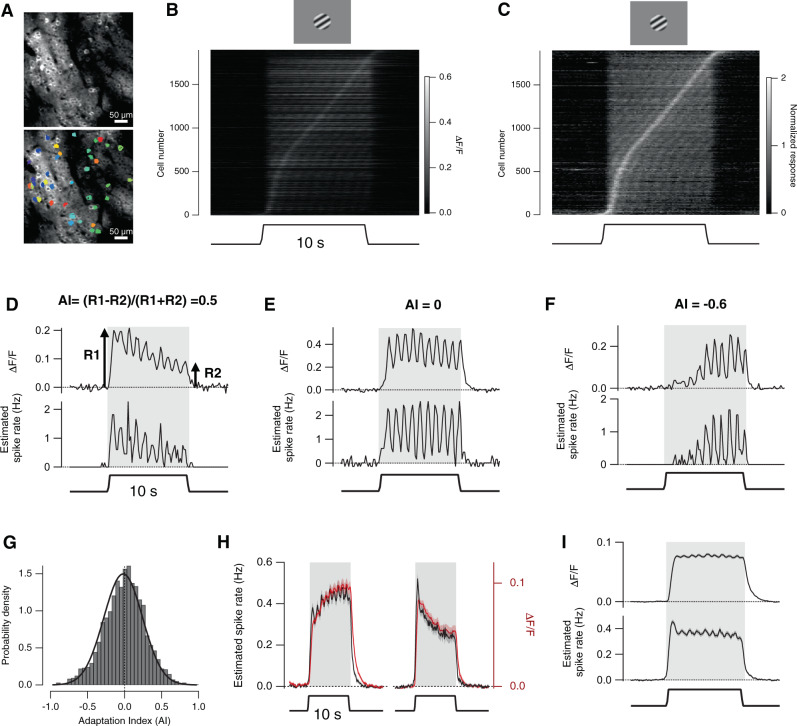
Opposite forms of plasticity in pyramidal cells. **A** Example of a field of view containing pyramidal cells (PCs) expressing GCaMP6f (top) and corresponding regions-of-interest (ROIs) defining individual neurons (bottom, see “Methods” section). **B** Raster plot showing responses of 1896 PCs to a high-contrast stimulus applied for 10 s (average of ten trials, collected from 18 mice). Image showed in **A** is an example of this population. **C** Results from **B** in which responses have been normalized relative to their peak amplitude and then sorted according to time of peak. Note that some cells generate their strongest response at the onset of the stimulus while others gradually sensitize. The phase shift between the stimulus and GCaMP signal was correlated with the adaptive index (AI) of the PC causing tilted bands to appear when responses were ordered this way. **D**–**F** Averaged responses of individual neurons measured with GCaMP (top) and then re-expressed as estimated spike rate (bottom), calculated using MLSpike algorithm. For each, an AI was calculated as (*R*1 − *R*2)/(*R*1 + *R*2) where *R*1 is the average response over the first 2 s of the stimulus and *R*2 the average response to the last 2 s. The AI of each neuron was 0.5 (**D** depressing adaptation, cell 150 in **C**), 0 (**E** no adaptation, cell 1031) and −0.6 (**F** sensitizing, cell 1613). **G** Distribution of the AI of 1896 PCs, calculated from GCaMP signals. The Gaussian fit is centered on AI = −0.019 with a sd of 0.26. A *t*-test and bootstrap test indicated that the mean AI was not significantly different from zero (*p* = 0.16, two-tailed *t*-test). **H** Comparison of GCaMP signals (red) and estimated spike rates (black) for PCs in the first 25% (left) and last 25% (right) of the distribution in **G**. Note the close correlation in the magnitude and direction of adaptive changes. **I** The averaged GCaMP signal (top) and estimated spike rate (bottom). Note the lack of contrast adaptation when the complete population of PCs is considered. Data are represented as mean ± SEM (shading area in **D**–**F**, **H**, **I**) or probability density (**G**). Source data are provided as a Source Data file.

Exposure to the high-contrast stimulus was followed by changes in activity that varied in both amplitude and direction across the population of PCs. Within a field of view, some neurons generated strong initial responses that then depressed gradually over the next 10 s. Other neurons generated weak initial responses but then increased their gain over a similar time-course (Fig. 1A–C). These opposite forms of plasticity represented either end of a continuum in the center of which many PCs did not show any significant adaptation to contrast. Individual examples of neurons that depressed, did not change gain and sensitized are shown in Fig. 1D–F.

To survey these adaptive effects across the population of PCs we quantified the strength and polarity of the change in response as an adaptive index (AI), defined as AI = (*R*1 − *R*2)/(*R*1 + *R*2), where *R*1 is the average response over the first two cycles of the 1 Hz stimulus and *R*2 is the average response over the last two cycles (Fig. 1D). We averaged responses over two cycles because of the 1 Hz modulation in the GCaMP signal reflecting the frequency of the stimulus. Neurons that depress therefore have an AI > 0, and those that sensitize an AI < 0. Surprisingly, as many PCs sensitized as depressed in response to the high-contrast stimulus and the distribution of AI across a population of 1896 neurons (*n* = 18 mice) was centered around zero (Fig. 1G). The GCaMP signal averaged over the first and last 25% of this distribution is shown in Fig. 1H (red traces) while the time-course of the response averaged across the complete distribution is shown in Fig. 1I (top). A mix of depressing and sensitizing adaptation was consistent over a series of stimulus trials in each field of view (Fig. S1) and between recording sessions in different mice (Fig. S2). The average AI had a weak tendency towards sensitization the closer the stimulus was to the center of the PCs receptive field (Fig. S3) but it did not depend on the orientation or direction of the test stimulus relative to the neuron’s preferred orientation (Fig. S4).

Might the appearance of sensitization in GCaMP signals be an artifact, perhaps reflecting slow clearing of Ca^2+^ ions and accumulation of Ca^2+^-bound reporter? Evidence against this idea is provided by the observation that the decay of the GCaMP signal at the end of the stimulus was not significantly different in depressing and sensitizing PCs (Fig. S5A, B). We also investigated the likely accuracy of AI measurements by using the MLSpike algorithm to reconstruct spiking activity that underlies the GCaMP6f signal^25–27^ (Fig. S6). The dynamics of the raw fluorescence signals agreed closely with the inferred spike rate in individual neurons (Fig. 1D–F) as well as with averages across sensitizing and depressing subsets (Fig. 1H). Further, the AI calculated from the inferred spike rate was proportional to that calculated from raw GCaMP6f signal with a correlation coefficient of 0.7 ± 0.03 (Fig. S7A) indicating that the raw GCaMP6f signal accurately reports adaptive changes in the spiking activity of PCs.

Experiments in anaesthetized animals have reported that depressing adaptation is dominant in V1^28,29^ but these results demonstrate that in awake mice there are two opposite forms of plasticity, depression and sensitization, that are roughly balanced to maintain stable levels of activity aross the population of PCs. A qualitatively similar combination of depressing and sensitizing responses has also been observed in macaque V1^12^.

### Sensitizing neurons preferentially signal a decrease in contrast

What is the functional role of these different forms of adaptation? It has long been recognized that a decrease in the gain of the response to a strong stimulus is useful to prevent saturation, allowing for continued signalling of any future increase in stimulus strength^30–32^. But such depressing adaptation also comes at the cost of a reduced sensitivity to a future *decrease* in stimulus strength^5,33^.

**Fig. 2:**
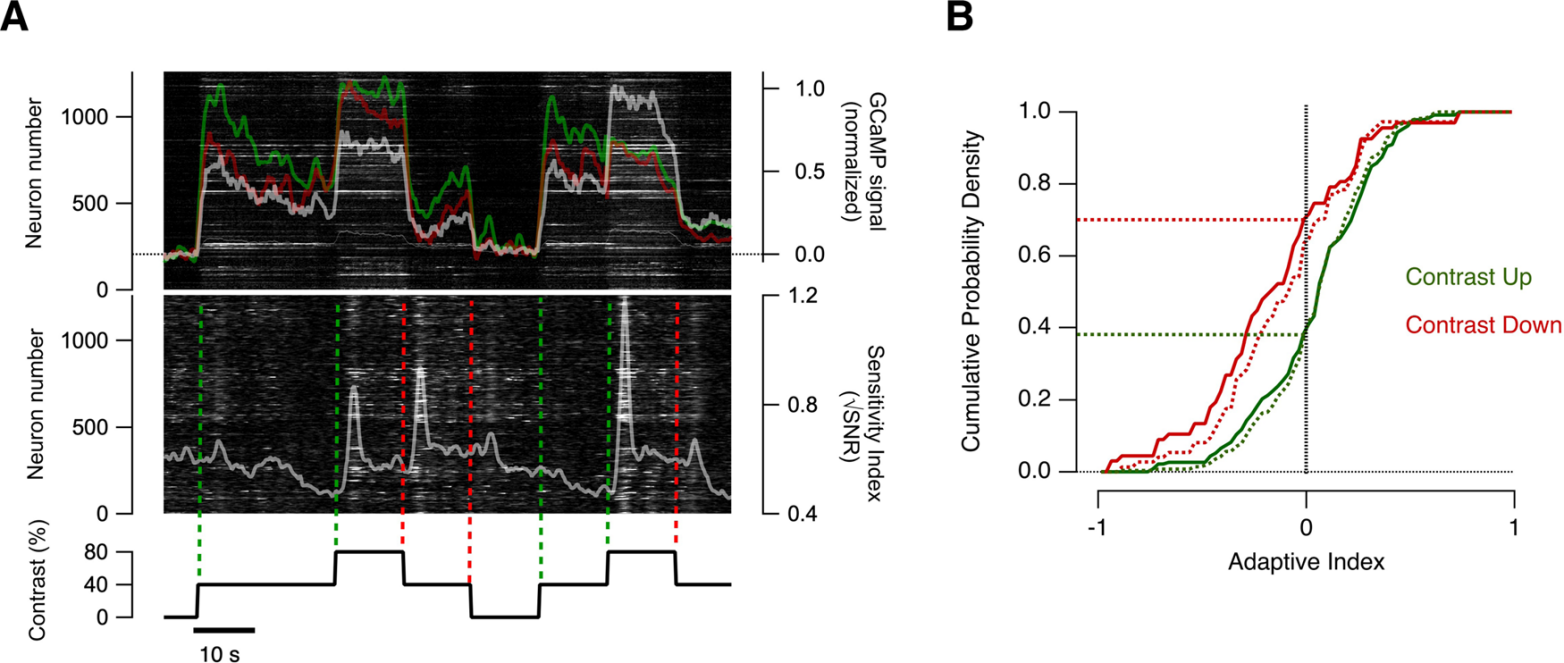
The majority of neurons detecting a decrease in contrast displayed sensitizing adaptation. **A** Raster plots showing the responses (top) and sensitivity index *d*′ (bottom) of 1261 PCs during presentation of a stimulus of changing contrast. The average is shown by the superimposed white traces. The green dashed lines indicate the contrast increases and the red line the contrast decreases. Setting a detection threshold of *d*′ > 1.41, 231 PCs detected either increase (18%, average response in green) while 67 detected the decrease (5%, average response in red). **B** The distribution of AIs of PCs detecting a contrast increase and decrease expressed as a cumulative density. Solid lines show the distribution for all transitions. Of PCs detecting the increase, 61% had an AI > 0, while 70% detecting the decrease had an AI < 0. The two distributions were different at *p* = 0.1 (Kolmogorov–Smirnov test). Dashed lines show this relation only for transitions from 40% contrast. In both cases, the AI was calculated from the transition from 40 to 80% contrast. Source data are provided as a Source Data file.

To assess how neurons of different AI contributed to signalling increases and decreases in contrast we applied signal detection theory^34^ and calculated the sensitivity index d’ at each time point during a stimulus of varying contrast as the square root of the signal-to-noise ratio (SNR)^35^ (see “Methods” section)${{{{{\boldsymbol{.}}}}}}$ Each contrast increase was 40%, from a baseline of either 0 or 40%, while each contrast decrease was 40% from a baseline of either 80 or 40%. The raster plot in Fig. 2A shows how *d*′ varied with time for a sample of 1261 PCs. The peak value of *d*′ was, on average, higher when the contrast increased compared to a decrease.

Setting a detection threshold of *d*′ > 1.41, 231 PCs detected either increase in contrast (18%) while 67 detected the decrease (5%). But what was the dominant form of adaptation in neurons signalling these changes? The cumulative distribution in Fig. 2B shows that PCs detecting the contrast increase were more likely to be depressing with 61% having an AI < 0. Conversely, PCs detecting a contrast decrease were much more likely to be sensitizing, 70% having an AI < 0. The picture that emerges is that neurons that sensitize in a high-contrast environment improve the detectability of future *decreases* in contrast.

### Distinct modes of adaptation in different classes of interneuron

What are the circuit mechanisms that can decrease the gain of some PCs while simultaneously increasing the gain of others? A key role for local inhibition is suggested by the observation that optogenetic activation of GABAergic neurons during a stimulus modifies subsequent adaptation^7^. We hypothesized, therefore, that PCs showing increases and decreases in gain might have different connectivities with local inhibitory circuits. To investigate this idea we began by assessing whether adaptive changes also occurred within different types of interneurons and found that they did.

**Fig. 3:**
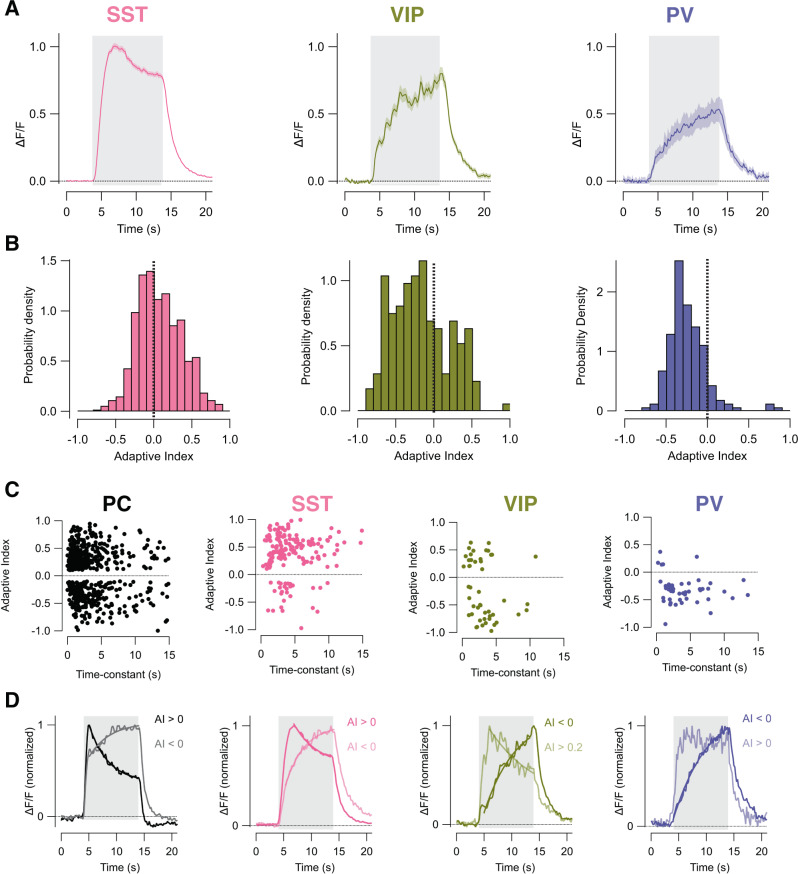
Adaptation in different classes of interneuron. **A** Average population response to the high-contrast stimulus in SST (left; four mice, 537 cells), VIP (middle, three mice, 175 cells) and PV interneurons (right, two mice, 162 cells). The timing of the stimulus is shown by the grey bar. **B** Distribution of AI measured within each interneuron type shown in **A**. Note the strongly sensitizing responses in PV and VIP interneurons. SST cells were predominantly depressing but some were sensitizing. **C** Scatter plots showing the AI of a neuron in relation to its time-constant of adaptation. From left: pyramidal cells (PC, *n* = 580), SST (*n* = 167), VIP (*n* = 48), and PV (*n* = 49) classes. The average time-constants for AIs > 0.1 were: 3.7 ± 3.4 s (PC, mean ± sd), 4.0 ± 3.1 s (SST), 2.8 ± 2.9 s (VIP), and 1.9 ± 2.7 s (PV). The average time-constants for AIs < −0.1 were: 3.1 ± 3.1 s (PC), 4.3 ± 2.3 s (SST), 3.5 ± 2.5 (VIP), and 4.3 ± 2.3 s (PV). The time-constant was only estimated if the major part of the response to the stimulus could be better described as a single exponential compared to a line and only if AI > 0.1 or AI < −0.1. **D** Estimates of the average time-constant of adaptation across populations of neurons were obtained by fitting exponential functions (smooth lines) to the average of their normalized responses. PC: AI > 0, 3.4 ± 0.2 s; AI < 0, 3.7 0.7 s. SST: AI > 0, 4.2 ± 0.4 s; AI < 0, 3.5 ± 0.3 s. PV: AI < 0, 6.4 ± 0.4 s. VIP: AI < 0, 7.5 ± 1.3 s; AI > 0.2, 8.5 ± 4.7 s. Note the similar time-constants of adapting and sensitizing adaptation in PC and SST (~3–4 s). In interneurons where sensitizing adaptation was dominant (VIP and PV), the time-constant was longer (~6–8 s). Data are represented as mean ± SEM (coloured shading area in (**A**). Source data are provided as a Source Data file.

Responses of interneurons expressing SST, VIP, or PV are shown in Fig. 3A with the corresponding distribution of AIs in Fig. 3B. Unlike PCs, sensitization to the high-contrast stimulus was the strongly dominant form of plasticity in both VIP and PV interneurons (average AI = −0.16 ± 0.03 and −0.24 ± 0.02; *n* = 175 and 162 cells, respectively). This predominance of sensitization in VIP and PV cells could not be explained by slower decay of the GCaMP signal compared to SST interneurons (Fig. S5C, D). SST interneurons were more heterogeneous with depression being dominant in 68% and only 32% showing sensitization (average AI = 0.07 ± 0.01, *n* = 537 cells). Within each cell type, there was no significant correlation between AI and the time-constant of adaptation, at least where a time-constant could be reliably measured (Fig. 3C). The time-constants of adapting and sensitizing adaptation were not significantly different when population responses were averaged in PC and SST (~3–4 s), as would be expected if there was a causal relation (Fig. 3D). In VIP and PV interneurons, where sensitizing adaptation was dominant, the time-constant was longer (~6–8 s; Fig. 3D), but the general picture is that adaptive effects occur over time-scales of a few seconds in all the major classes of neuron in layer 2/3 of V1.

### PV interneurons drive depressing adaptation

**Fig. 4:**
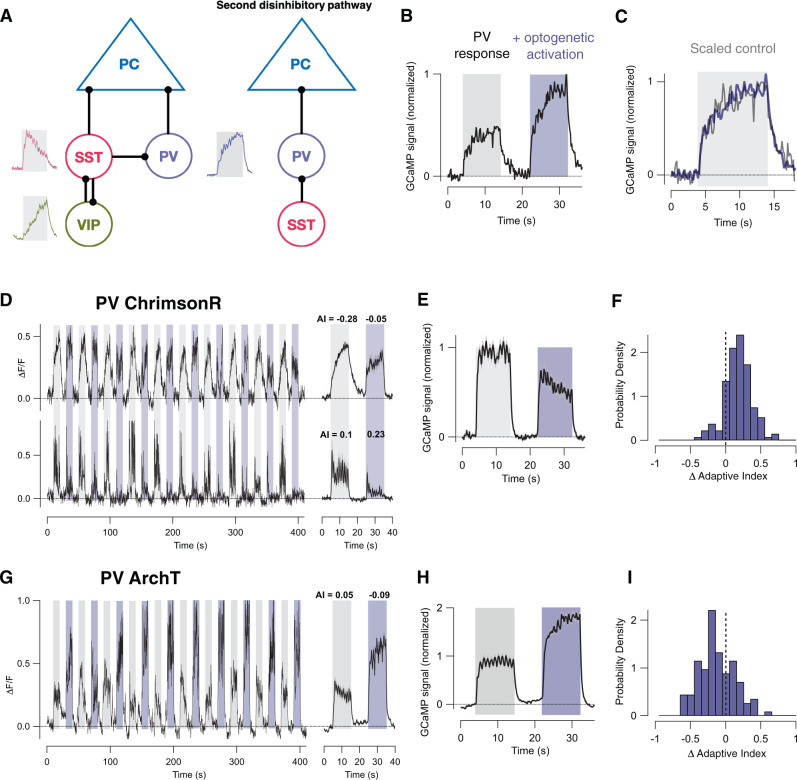
PV interneurons drive depressing adaptation in pyramidal cells. **A** Schematic of major inhibitory connections in layer 2/3 of V1. Left: major direct inputs to PCs are from SST and PV interneurons. VIP→SST pathway disinhibits PCs. Right: A second disinhibitory pathway from SST→PV. Average response trace from Fig. 3 also shown. **B** Population average of GCaMP6f signal in PV cells in response to the visual stimulus delivered without (grey bar) and with (purple bar) optogenetic activation using ChrimsonR (89 cells, two mice). **C** Control response (grey) scaled by a factor of 1.79 demonstrating the same time-course as the response during activation of ChrimsonR (purple). **D** An example of the responses of PCs when optogenetically activating PV interneurons. Left: average responses from two fields of view recorded from the same mouse on the same day (top, 18 cells; bottom, 6 cells). Right: average response from these trials. Top: AI = −0.28 ± 0.03 in control conditions and −0.05 ± 0.03 during PV activation (difference significant at *p* = 0.002, WSR test). Bottom: AI = 0.13 ± 0.04 in control conditions and 0.21 ± 0.07 during PV activation (no significant difference, WSR test). **E** Average of paired stimulus trials from experiments shown in **D**; *n* = 3 mice, 296 cells). Over-activation of PV cells reduced gain in PCs and pushed the population towards depressing adaptation. **F** Distribution of changes in AI caused by activation of PV cells expressing ChrimsonR. The distribution is shifted towards positive values (significant at *p* = 0.0001, WSR test). **G** An example of the response of PCs when optogenetically inhibiting PV interneurons with ArchT (purple bars). Average of 43 cells in one FOV. AI = 0.04 ± 0.05 in control conditions and −0.09 ± 0.05 during PV inhibition (difference significant at *p* = 0.03; WSR test). **H** Average of paired stimulus trials from experiments shown in **G** (*n* = 3 mice, 264 cells). **I** Distribution of changes in AI caused by inhibition of PV cells expressing ArchT. (significant at *p* = 0.002; WSR test). Data are represented as mean ± SEM (grey shading area in **B**, **E**, **H**) or probability density (**F**, **I**). Source data are provided as a Source Data file.

Optogenetics was used to test for causal links between interneuron activity and adaptive effects in PCs. The strongest inhibitory connections within Layer 2/3 are shown in Fig. 4A, based on electrical recordings, optogenetics and viral tracing^36–39^. We began by investigating the role of PV interneurons which are the most numerous inhibitory subtype and have been shown to modulate the gain of visual responses in PCs^40^. PV interneurons are also relatively isolated within inhibitory networks of the cortex, establishing strong and *direct* connections with PCs but not transmitting significantly to SST or VIP interneurons^37^, thereby making it easier to interpret their role in adaptation.

First, we expressed both ChrimsonR^41^ and GCaMP6f in PV cells to assess how increased excitation altered the dynamics of their response to the high-contrast stimulus. At an illumination power of 60 µW, the activity of the PV cells during the delivery of the high-contrast stimulus was scaled up by an average factor of 1.78, without a significant change in time-course of sensitization (Fig. 4B, C; *n* = 89 cells).

To test how over-activating PV interneurons in this way impacted on the activity of PCs expressing GCaMP6f we used interleaved trials of the stimulus with and without simultaneous co-activation of ChrimsonR (Fig. S8A, B). Averaged responses in two fields of view are shown in Fig. 4D, one which sensitized in control conditions (top, AI = −0.28, 18 PCs) and one that displayed little adaptation (AI = 0.1; 6 PCs). Increasing PV activity (purple bars) had two basic effects: the initial gain of the response was reduced and the AI increased, as can be seen in the response averaged over 296 PCs (Fig. 4E). The distribution of changes in AI caused by over-activation of PV cells is shown in Fig. 4F and is shifted towards positive values (significant at *p* < 10^−4^, WSR test). Such changes in the AI of PCs caused by optogenetic activation of interneurons were not correlated with the initial amplitude of the response measured under control conditions (Fig. S9) and were rapidly reversible (Fig. S10).

To test whether *normal* levels of activity in PV interneurons also promote depressing adaptation in PCs we used ArchT to inhibit PV activity (Fig. 4G). Reducing PV activity increased the average amplitude of the response in PCs (Fig. 4H) and caused sensitization to predominate (Fig. 4I, *n* = 264 cells, significant at *p* = 0.001). Together, the results in Fig. 4 demonstrate that the activity of PV interneurons both reduces the gain of visual responses in PCs and promotes the depressing mode of adaptation.

### SST interneurons drive sensitization through two distinct mechanisms

**Fig. 5:**
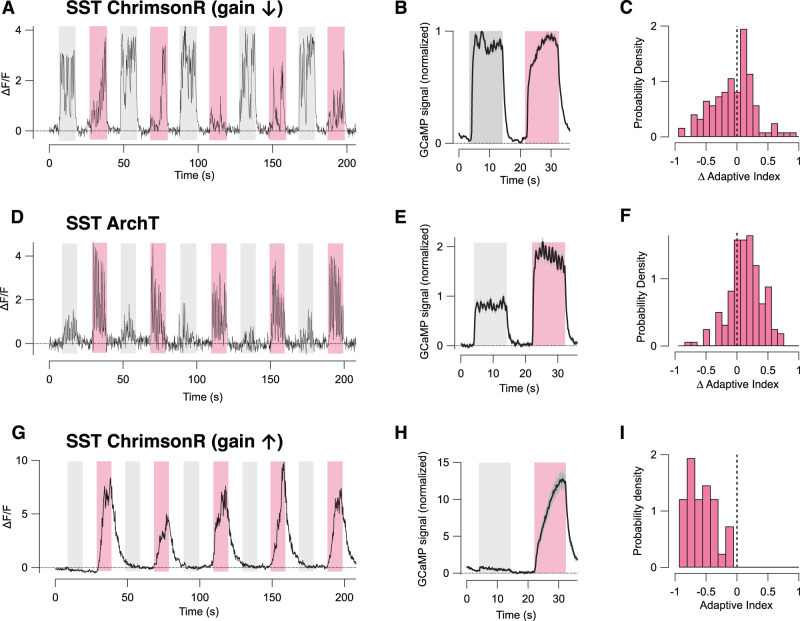
SST interneurons drive sensitization of pyramidal cells. **A** An example of the response of a PC when optogenetically activating SST interneurons expressing ChrimsonR. Stimulus trials with LED illumination (pink bars) and without (grey bars) were interleaved. Increasing activity of SST cells decreased the initial gain, which was followed by sensitization. **B** Average of paired stimulus trials from experiments shown in **A** (*n* = 3 mice, 552 cells). **C** The distribution of changes in adaptive index of PCs during SST cell activation. The shift towards negative values of AI (sensitization) was significant at *p* = 10^−5^ (WSR test). **D** An example of the response of a PC when optogenetically inhibiting SST interneurons expressing ArchT (pink bars). **E** Average of paired stimulus trials from experiments shown in **D** (*n* = 3 mice, 319 cells). **F** The distribution of changes in AI of PCs during SST cell inhibition. The shift towards positive values of AI (depression) was significant at *p* = 0.02 (WSR test). **G** An example of responses from one of the subset of PCs (12% of total) that was not normally significantly responsive to the stimulus, but became strongly responsive on overactivating SST interneurons expressing ChrimsonR. **H** Average of paired stimulus trials from experiments illustrated by G (*n* = 3 mice, 67 cells). In these PCs sensitization was correlated with a dramatic increase in gain, indicating that SST cells were exerting a disinhibitory effect. **I** The distribution of AI in PCs that became responsive during SST excitation through ChrimsonR: these were uniformly sensitizing (average AI = −0.64 ± 0.22). Data are represented as mean ± SEM (grey shading area in **B**, **E**, **H**) or probability density (**C**, **F**, **I**). Source data are provided as a Source Data file.

After PV interneurons, the second major inhibitory input to pyramidal cells originates from the SST subtype, which can themselves be modulated by VIP interneurons^36,42^ (Fig. 4A). Optogenetic activation of SST interneurons (Fig. S11A) had distinct actions on different PCs and a simple way to distinguish these was to separately consider the 88% of PCs that responded to the stimulus under normal conditions (Fig. 5A–C) and the 12% that did not (Fig. 5G–I). In the first group, the dominant effect of overactivating SST interneurons was to decrease the initial gain of the response to stimulus onset, consistent with their receiving direct inhibitory inputs from SST interneurons (Fig. 5A, B). Simultaneously, the adaptive effect across the population of PCs shifted towards sensitization (Fig. 5C; significant at *p* = 10^−5^, WSR test, *n* = 552 cells). Conversely, suppressing SST interneurons expressing ArchT (Fig. S11B) increased the initial response by a factor of 2.22 ± 0.10 (Fig. 5D, E), and the PC population shifted towards depressing adaptation (Fig. 5E, F; *n* = 319 cells; significant at *p* = 0.02, WSR test).

The smaller subset of PCs (12%) only responded when the stimulus was paired with overactivation of SST interneurons and these uniformly underwent sensitizing adaptation once active (Fig. 5G–I; average AI = −0.39 ± 0.03, *n* = 67 cells). These results lead us to propose two mechanisms by which SST interneurons drive PCs to sensitize, one associated with a decrease in the initial gain (Fig. 5A–C) and the second with a dramatic increase (Fig. 5G–I). Below we present evidence that these differing actions reflect whether the PC is influenced by SST interneurons directly, or indirectly by a disinhibition pathway through PV interneurons.

The general picture emerging from the results in Figs. 4 and 5 is that bidirectional manipulations of activity in PV and SST interneurons push adaptive effects within the PC population from one polarity to another. This in turn suggests a model that explains variations in adaptive properties of PCs as reflecting differences in the balance between SST inputs driving sensitization and PV inputs driving depression. This model is explored further below.

### VIP interneurons drive depressing adaptation

**Fig. 6:**
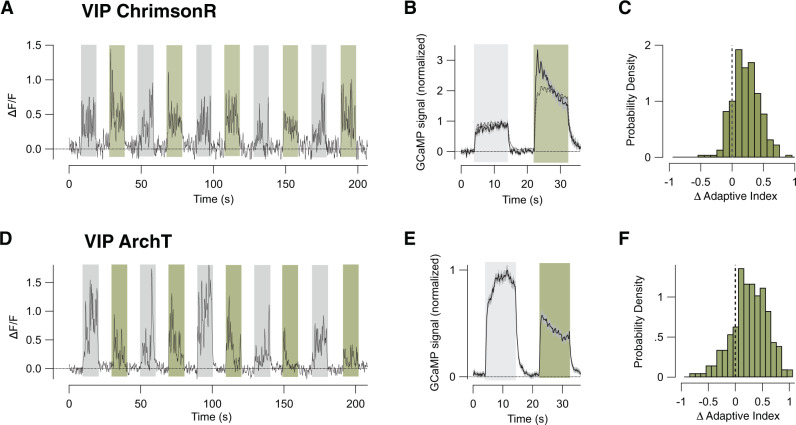
VIP interneurons drive sensitization of pyramidal cells. **A** An example of the response of a PC when optogenetically activating VIP interneurons expressing ChrimsonR. Stimulus trials with LED illumination (green bars) and without (grey bars) were interleaved. Increasing activity of VIP cells increased the initial gain of PCs, demonstrating effective disinhibition, which was followed by depressing adaptation. **B** Average of paired stimulus trials from experiments shown in **A** (*n* = 3 mice, 414 cells). The solid trace shows the effect of 1 mW illumination power and the dashed trace 0.06 mW. Note that the lower level of photoactivation caused a smaller increase in the initial amplitude of the response and a smaller shift to depressing adaptation. **C** The distribution of changes in adaptive index in PCs during VIP cell activation. The shift towards positive values (depression) was significant at *p* = 10^−6^ (WSR test). **D** An example of the response of a PC when optogenetically inhibiting VIP interneurons expressing ArchT. **E** Average of paired stimulus trials from experiments shown in **D** (*n* = 3 mice, 421 cells). **F** Distribution of changes in AI of PCs during VIP neuroninhibition. The distribution was shifted towards depressing adaptation (significant at *p* = 10^−7^; WSR test). Data are represented as mean ± SEM (grey shading area in **B**, **E**) or probability density (**C**, **F**). Source data are provided as a Source Data file.

We turn now to the third major class of inhibitory interneuron expressing VIP. Direct connections between these and PCs are weak but they exert powerful indirect actions through SST expressing neurons^43,44^. This VIP→SST disinhibition pathway regulates network dynamics in V1 during changes in behavioural state, such as engagement in locomotion or arousal^18–21^. Is the VIP→SST disinhibition pathway also involved in adaptation? Consistent with this idea, overactivation of VIP cells had the same general effect as inhibition of SST cells, increasing the initial gain of PCs and shifting the population towards depressing adaptation (Fig. 6A–C). When the power of the amber exciting beam was 1 mW, the initial response in PCs increased by 200% (solid line in Fig. 6B) and the distribution of the change in AI in PCs was simultaneously shifted towards depressing adaptation (Fig. 6C; *n* = 414 cells; significant at *p* < 10^−6^, WSR test).

To test whether normal levels of activity in VIP interneurons were sufficient to exert adaptive effects in PCs, we inhibited their response to the stimulus using ArchT (Fig. 6D–F). An illumination power that reduced the initial response of PCs by an average factor of 61% caused a simultaneous shift in AI towards depression (Fig. 6F; *n* = 535 cells; significant at *p* = 10^−7^, WSR test). This result appears paradoxical at first; over-activating or inhibiting VIP interneurons both push the population of PCs towards depression. A resolution likely lies in the observation that SST interneurons were a mixed population of depressors and sensitizors (Fig. 3). In comparison, VIP interneurons were more uniformly sensitizing indicating that they modulate the depressing subset of SST cells. Reducing VIP activity can therefore be expected to shift the SST population as a whole towards sensitization, thereby driving PCs towards depression. This explanation is consistent with conceptual models that are tested further below.

### Locomotor behaviour increases gain while maintaining the balance of adaptive effects

**Fig. 7:**
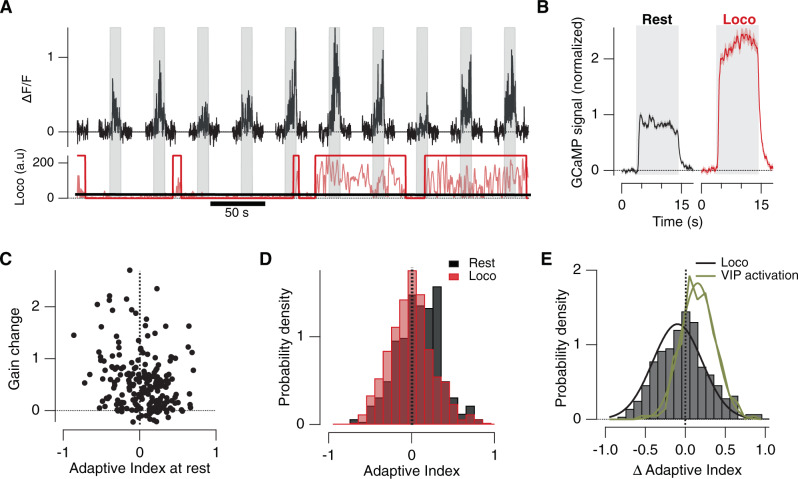
Locomotion increases the gain of both depressing and sensitizing pyramidal cells. **A** An example of the responses from a single PC (top trace) to ten stimulus presentations (grey bars). Below is shown the output from the locomotion monitor (noisy red trace), which was binarized by setting a threshold (black line). A trial was counted as occurring at rest if there was no coincident locomotion and only counted as locomotion if this was continuous during both presentations of the stimulus. **B** Averaged responses at rest and during locomotion, normalized to the peak response at rest (*n* = 253). **C** The relative increase in response amplitude during locomotion as a function of the AI at rest (each point from one PC). The gain increase was not significantly correlated with the AI at rest (correlation coefficient −0.14 ± 0.18). Both depressing and sensitizing neurons experienced increases in gain during locomotion. **D** The distribution of AI measured during trials at rest (black; mean AI = 0.028 ± 0.014; *n* = 324) and during locomotion (red; mean AI = −0.06 ± 0.012; *n* = 512). The mean AI was not significantly different from zero in either state (*t*-test). **E** The distribution of the change in AI caused by a transition from rest to locomotion (grey bars) can be described by a Gaussian centered at ΔAI = −0.08 with s.d = 0.31. The shift towards negative values (sensitization) was significant at *p* = 0.001 (*t*-test, two-tailed). The effects of optogenetically activating VIP interneurons (which also increased the gain of the response) are compared (green, from the distribution in Fig. 6C). The Gaussian describing the effects of this manipulation is centered at ΔAI = 0.15 with s.d = 0.22. The shift towards positive values (depression) was significant at *p* = 0.0001 (*t*-test, two-tailed). Data are represented as mean ± SEM (grey and red shading area in **B**) or probability density (**D**, **E**). Source data are provided as a Source Data file.

Dramatic increases in the gain of responses in V1 occur when an animal transitions from “rest” to an “active” or aroused state signaled by locomotion^20,21,44^. The VIP→SST disinhibition pathway plays a key role in this gain increase^18,19^ but also drives PCs towards depression (Fig. 6A–C). Does locomotion therefore also shift the population of PCs towards depressing adaptation? To investigate this question we compared visual responses measured at rest and during locomotion. Figure 7A shows an example PC recording in which the mouse transitioned from rest to a locomotory state, increasing the response of PCs to the stimulus. Averaged responses in a sample of 449 PCs are shown in Fig. 7B, where locomotion increased the peak response by an average factor of 1.45 ± 0.18. Notably, this change in gain was not correlated with the AI of the PC at rest (Fig. 7C). The distribution of AI at rest and during locomotion are shown in Fig. 7D: the means did not differ significantly from zero in either state, indicating that depressing and sensitizing PCs remained in approximate balance. Locomotion therefore increased the gain of visual responses across the population of PCs expressing different forms of adaptation.

**Fig. 8:**
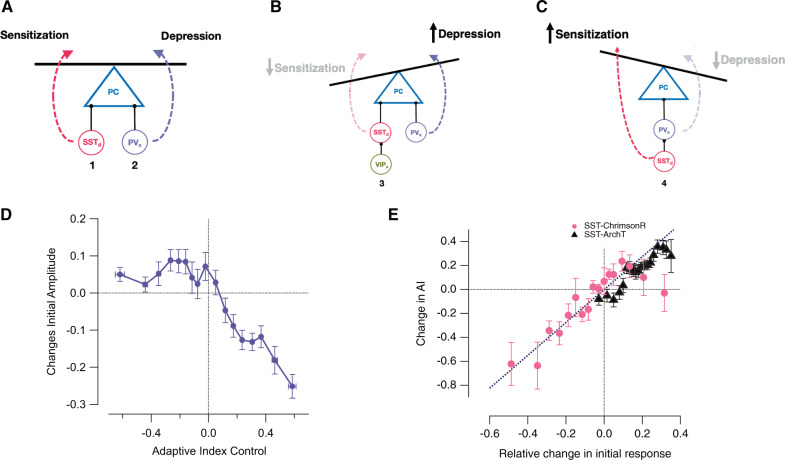
Local circuits controlling depressing and sensitizing adaptation. **A** conceptual model consistent with the results in Figs. 1–5. The adaptive response in PCs varies because of differences in the balance between direct inputs from SST and PV interneurons. PV_s_ interneurons were sensitizing and therefore drive depression. The majority of SST cells were depressing (denoted SST_d_) and drive sensitization. **B** Activity in VIP interneurons favoured depression (Fig. 6), consistent with reducing the sensitizing input of SST interneurons through the VIP_s_→SST_d_ disinhibition pathway. **C** An additional SST→PV_s_ disinhibition circuit is proposed to account for the subset of not responsive PCs with a dramatic increase in gain when activating SSTs (~12%) (Fig. 5G–I). **D** A test of the model in **A** focusing on PV interneurons. The plot shows the relation between the change in initial amplitude in PCs upon activation of PV interneurons (measure of direct PV input) and the AI under control conditions. PCs receiving the strongest PV input displayed the strongest depressing adaptation. For sensitizing PCs (AI < 0), PV activation had little effect on the initial amplitude of the response to the high-contrast stimulus, indicating that these received weak PV input. **E** A test of the model in **A** focusing on SST interneurons. The plot shows the relation between the change in AI of PCs caused by an optogenetic manipulation and the relative change in initial amplitude of the response (measure of direct SST input). Pink circles show effects of activating SST interneurons using ChrimsonR: the stronger the SST input the stronger the sensitization (Correlation coefficient, *r* = 0.85). Black triangles show effects of inhibiting SST interneurons using ArchT. The greater the increase in gain, the stronger the shift to the depressing mode of adaptation (Correlation coefficient, *r* = 0.93). Dashed line: fit constrained to pass through the origin. These results support the prediction of the model in **A**, that PCs with the strongest direct input from SST cells will also have the strongest tendency to sensitize. Data are represented as mean ± SEM of 20–30 PCs per point. Source data are provided as a Source Data file.

The distribution of the change in AI associated with the transition from rest to locomotion is shown in Fig. 7E, where it is compared with the effects of optogenetically activating VIP interneurons (results from Fig. 6C). Selective activation of VIP interneurons caused a smaller increase in gain of PCs (101 ± 5%), but nonetheless significantly shifted the population towards depressing adaptation. These results indicate that locomotion does not increase the gain of PCs exclusively through the VIP→SST disinhibition pathway. Rather it seems that locomotion also increases the gain of PCs through a circuit that drives sensitization. The best candidate for this is the SST→PV disinhibition pathway uncovered by activating SST interneurons using ChrimsonR (Figs. 5G–I and 8C below).

### Circuits determining the balance between depression and sensitization

Graphical models of the inhibitory circuits that control opposite forms of adaptation in PCs are shown in Fig. 8A–C. These begin with current understanding of the basic connectivity of inhibitory interneurons in V1^36,37,45^ on which we have superimposed the network dynamics measured in normal conditions and during various optogenetic manipulations (Figs. 1 and 3–6). We propose that the adaptive response in different PCs vary because of differences in the balance between direct inputs from SST and PV interneurons (circuits 1 and 2 in Fig. 8A). PV cells are uniformly sensitizing (Fig. 3) and therefore drive depression (Fig. 4), while the majority of SST cells are depressing and drive sensitization (Fig. 5). Activity in VIP interneurons also favours depression (Fig. 6), consistent with reducing the sensitizing input of SST interneurons through the VIP→SST disinhibition pathway (circuit 3 in Fig. 8B).

A key prediction of the “balance” model depicted in Fig. 8A is that PCs with the strongest tendency to depress also receive the strongest input from PV cells. We tested this prediction by separating PCs according to their AI measured under control conditions and then assessing how effectively they were inhibited by optogenetic activation of PV cells expressing ChrimsonR. Figure 8D demonstrates that, as predicted by the model, the more strongly a pyramidal cell depressed (AI > 0), the stronger the inhibitory input it received from PV interneurons. In contrast, the initial gain of sensitizing PCs (AI < 0) was far less sensitive to PV activation, indicating that these neurons received weak PV input.

A second prediction of the”balance” model is that PCs with the strongest direct input from SST cells will have the strongest tendency to sensitize, and this was confirmed by the analysis in Fig. 8E. Here, we grouped PCs according to the change in gain caused by optogenetic activation of SST interneurons, assessed from the initial amplitude of the response to the high-contrast stimulus. The change in AI for these different groups is plotted as the pink circles and it can be seen that the stronger the SST input the stronger the sensitization (correlation coefficient, *r* = 0.85). A similar analysis was carried out when SST interneurons were inhibited through ArchT (black triangles in Fig. 8E). The greater the increase in gain, the stronger the shift to the depressing mode of adaptation (correlation coefficient, *r* = 0.93), indicating that SST inputs were normally driving sensitization.

The balance model does not account for the 12% of PCs that were initially silent but then displayed a dramatic increase in gain when over-activating SST interneurons (Fig. 5G–I). Clearly, these PCs do not receive significant direct input from SST cells, which must instead act in a disinhibitory mode. This is most likely to occur through PV cells (Fig. 8C) given that these provide strong inhibitory inputs to all PCs^36,37^ and an SST→PV disinhibition pathway has been shown to regulate visual responses in V1^38,39^. Based on these various observations we propose that SST interneurons can drive sensitization in at least two distinct groups of PCs, one associated with a decrease in initial gain (Fig. 5A) and the second with an increase (Fig. 5G).

## Discussion

This investigation demonstrates that pyramidal cells in layer 2/3 of V1 adapt to a high-contrast stimulus to varying degrees and with opposite forms of plasticity. While many PCs initially respond with high gain followed by gradual depression over periods of several seconds, these are balanced by cells that respond with low gain but then sensitize (Fig. 1). These different modes of adaptation reflect the dynamics of signals in three classes of interneuron (Fig. 3), and a variety of optogenetic manipulations were consistent with a model in which the net adaptive effect reflects the balance between direct inputs from PV interneurons, driving depression, and a subset of SST interneurons, driving sensitization (Figs. 4–8). A second level of control occurs through disinhibitory circuits, the VIP→SST pathway driving depression (Fig. 6) and the SST→PV pathway driving sensitization (Fig. 5). We also provide evidence that long-range inputs active during locomotion drive both disinhibitory circuits (Fig. 7). The general picture is that different subsets of PCs preferentially signal increases or decreases in contrast (Fig. 2), but all are normally engaged to maintain the balance between different forms of adaptation across the whole population of PCs (Figs. 1 and 7).

### What is the functional role of opposite forms of adaptation?

Within natural images, pixels nearby in space and/or time tend to be correlated and a general principle in understanding the design of sensory systems is the need to remove such redundancies from the neural signal so that information can be transmitted more efficiently^46,47^. Depressing adaptation fulfills this function by suppressing responses to aspects of the sensory stream that are over-represented, increasing the dynamic range available for signaling deviations from the local image statistics^31,32^. In the visual system, adaptation as a decrease in sensitivity can occur for responses driven by a number of properties of a visual stimulus, including mean luminance and temporal and spatial deviations from the mean (contrast)^7,48^, as well as features such as the orientation of edges^13,49^ or object motion^50^.

It is relatively recently that sensitization has been identified as an important adaptive mechanism in vision^32^ being observed using different stimulus paradigms leading to different proposals for its function. In macaque V1, shifts in orientation tuning of neurons *towards* the orientation of an adapting stimulus can be caused when (suppressive) normalizing inputs are activated in the surround of a neuron’s receptive field and then undergo depression. The resulting increase in responsivity within the receptive field center then causes a stronger response to a similar orientation newly appearing in that location, improving the ability to spatially segregate that stimulus from the background. Such segregation has been suggested to underlie the interactions between adaptation and attention measured using psychophysics^32,51^.

In mouse V1, sensitization becomes the dominant form of contrast adaptation when the mouse is conditioned to a reward associated with the stimulus; when the stimulus is not behaviourally relevant, the dominant form of adaptation is depressing^11^. These observations lead to the suggestion that the balance between adaptation as increases and decreases in gain is modulated by top-down attentional mechanisms to enhance the signalling of stimuli that have become behaviourally important. Changes in behavioural state can therefore alter adaptation to a consistent visual stimulus, indicating that the efficiency of neural coding should be considered not only in relation to the statistics of the external world but also in relation to the animals’ behavioural priorities. The behavioural change that we have investigated, transitions between rest and locomotion, is much more acute than conditioning to a reward and in this case depressing and sensitizing responses remained in balance despite the large increase in average population activity.

Opposing forms of adaptation have also been observed in the retina. While some bipolar cells and ganglion cells depress after the initial response to an increase in contrast, others respond weakly at first but then sensitize^5,33,52^. The particular contribution of sensitizing neurons is to maintain retinal sensitivity to future decreases in contrast to be signalled more effectively, improving the overall rate of information transfer through the optic nerve when the contrast fluctuates^33,52^. It may be that opposing forms of plasticity in V1 also provide a general strategy by which the population of PCs “hedge bets” in the face of an unpredictable future, and this idea was supported by the observation that sensitizing PCs played the major part in signalling a decrease in contrast (Fig. 2).

The balance between opposite forms of adaptation may also vary in different visual pathways. In the retina of primates, for instance, a high-contrast stimulus of low spatial frequency causes depressing adaptation in large parasol ganglion cells but sensitization in midget cells in the fovea^52^. Similarly, motion of the background mimicking eye movements reduced contrast sensitivity in parasol cells but increased sensitivity in midget cells, leading to the suggestion that sensitization specific to the fovea “primes” the retina to signal information about an object fixated on immediately after eye movement. This process has a direct psychophysical counterpart in humans: increased contrast sensitivity immediately following eye movements^53^. It will be interesting to explore whether other functional properties of sensitizing and depressing PCs in V1 also differ.

### Circuit mechanisms of depressing and sensitizing adaptation

In the retina, both depressing and sensitizing adaptation begin in the synaptic compartments of bipolar cells that transmit the visual signal to ganglion cells. At this locus, the major cause of depressing adaptation is depletion of vesicles available for release, while sensitization reflects depression in inhibitory amacrine cells providing GABAergic feedback^5,24,52,54^. Although sensitization in V1 could in principle also be driven by facilitation of excitatory inputs, current evidence indicates that reduced inhibition is the driver of sensitization in PCs, both to orientation^12,55^ and contrast (Figs. 5D–F and 8E). It will nonetheless be worthwhile investigating how modulation of excitatory inputs to V1 might also contribute to contrast adaptation in PCs. Inputs from thalamocortical neurons are known to undergo activity-dependent depression, although the time-scales are of the order of hundreds of milliseconds^56–58^ rather than the several seconds over which we observe contrast adaptation in PCs.

The issue of stimulus time-scale may also be important in understanding why we did not observe any significant correlation between the orientation/direction preference of the PC and the adaptive index of the response to the adapting stimulus presented at other orientations (Fig. S4). If the adaptor is presented on the time-scale of hundreds of milliseconds, surround suppressive effects depend on orientation and can retune PCs to prefer orientations away from the adapting stimulus^12,13,30^, while a stimulus presented over ~40 s will instead retune some neurons towards the adaptor orientation^55^. Here, we used adapting stimuli presented for 10 s but it is clear that different adaptative effects can occur over varying time-scales^32,55^, and it will be interesting to assess how far opposite forms of adaptation depend on stimulus duration.

It will be equally important to understand how far the plasticity of excitatory transmission contributes to adaptation in interneurons. Paired electrical recordings have shown that while local PC→VIP connections tend to depress during 50 Hz stimulation, PC→SST connections tend to facilitate^59^. If these different forms of presynaptic plasticity operate during normal levels of visually-drive activity they would be expected to cause sensitization in VIP interneurons and depression in SST interneurons, and these were indeed the dominant forms of adaptation observed in these two classes (Fig. 3). A better understanding of the role of excitatory inputs during adaptation might come with in vivo imaging of glutamate release using iGluSnFR^60^ or presynaptic calcium signals using SyGCaMPs^17,61^. It will be just as important to investigate the possibility that inhibitory synapses also show use-dependent changes in gain.

### Some future directions

Here, we have investigated adaptive changes in V1 under a very limited set of stimulus conditions testing responsivity to contrast but processing in V1 also adapts to higher-order statistics of a stimulus, such as spatial correlations forming an orientated edge^4^. Adaptation to orientation also involves simultaneous increases and decreases in sensitivity of different pyramidal cells such that the average activity across the population is maintained despite a fluctuating input^13^. Are the inhibitory circuits adjusting sensitivity to contrast also involved in adaptation to orientation or other properties of the visual input?

We also need to understand how adaptation to external stimuli interacts with other changes in behavioural or cognitive state, such as those associated with spatial attention or conditioning to a reward stimulus. Sensitization, for instance, becomes the dominant form of contrast adaptation when a mouse is conditioned to a rewarded stimulus^11^ and we now need to understand how changes in the inhibitory circuits regulating the activity of PCs relate to such behavioural changes, as well as the role of top-down inputs interacting with these circuits^62–64^.

The results we have presented outline the role of inhibitory microcircuits in adaptation to a fundamental feature of a visual stimulus—contrast—providing a more detailed framework for investigating how external stimuli and internal state interact to adjust processing in V1.

## Methods

### Experimental model and subject details

All experimental procedures were conducted according to the UK Animals Scientific Procedures Act (1986). Experiments were performed at University of Sussex under personal and project licenses granted by the Home Office following review by the Animal Welfare and Ethics Review Board.

Experiments were performed on 27 adult transgenic mice of either sex (4–10 months old) expressing the Cre recombinase in specific subsets of interneurons on a C57BL/6J background. Results are reported from nine VIP-Cre mice (VIP tm1(cre)Zjh/J Jackson #010908), eight PV-Cre (Pvalb tm1(cre)Arbr/J, Jackson #008069), and ten SST-Cre (SST tm2.1(cre)Zjh/J, Jackson #013044). Mice were housed individually on inverted light-dark cycles and had access to a complex enriched environment after the end of each imaging session. This environment was large (~80 × 40 × 40 cm) and contained a number of toys, platforms and tubes that encouraged motor activity. As a result, mice were engaged in running the large majority of the time during an imaging experiment.

### Animal preparation and virus injection

We prepared mice for multiphoton imaging of the visual cortex following established protocols^65^. Surgeries were performed on adult mice (P60-P90) in a stereotaxic frame under isoflurane anaesthesia (induction at 4% and 1–2% during surgery). A titanium head plate was attached to the skull, followed by a 3 mm craniotomy and durotomy to expose the brain. We then injected different combinations of viruses for imaging and manipulating cellular activity. To image calcium activity in pyramidal cells we used AAV1.CaMKII.GCaMP6f.WPRE.SV40 (*n* = 12 mice, titer 4 × 10^11^ GC/ml) or AAV5.CaMKII.GCaMP6f.WPRE.SV40 (*n* = 6 mice, titer 4 × 10^11^ GC/ml) viruses. To image calcium activity in interneurons we expressed AAV9.CAG.Flex.GCaMP6f.WPRE.SV40 in Cre lines (PV-Cre, VIP-Cre, SST-Cre, *n* = 9 mice, titer 2 × 10^12^ GC/ml). To excite interneurons optogenatically we used rAAV9/Syn.Flex.ChrimsonR.tdTomato (*n* = 12 mice, 2 × 10^12^ GC/ml) and to inhibit we used AAV5.CBA.Flex.ArchT-tdTomato.WPRE.SV40 (*n* = 10 mice, titer 2 × 10^12^ GC/ml). Viruses were injected with a beveled micropipette attached to a stereotaxic micromanipulator. Injections were performed at a single site in monocular V1 (2.4–2.8 mm lateral and 3.5–4.0 mm posterior from Bregma) and at 250–350 µm depth. A volume of 1 µl of virus was injected at a single site, at a speed of 20–50 nl/min and the micropipette was retracted after a 15–20 min waiting period. A cranial window assembly was made of 2 round coverslips, (3 mm diameter, thickness #1) bonded to each other and then to a 5 mm round coverslip, (thickness #1) using optical glue (ThorLabs NOA63). The window was then placed in the craniotomy and sealed with Vetbond and dental cement. Mice were returned to their home cage for 2 weeks following surgery. At least 1 week before imaging started mice were habituated to head fixation under the microscope, as well as the spherical treadmill. The two LED monitors were turned on (grey screen and occasionally visual stimuli). Imaging of neural activity began 3–5 weeks after surgery and injection.

### Muiltiphoton imaging in vivo

Fluorescence was measured with a two-photon microscope (Scientifica SP1, galvonometer mirrors) controlled using Scanimage 5 software (Vidrio Technologies) and using a Nikon 16× water-immersion objective (0.8 NA). The field of view was 630 μm wide. The framerate was set at 6.07 Hz in bidirectional mode, for an image resolution of 256 by 200 pixels and data acquired with a 250 MHz digitizer (National Instruments). The light source was a Ti:sapphire laser (Chamelon 2, Coherent), tuned to 940 nm. Laser power under the objective never exceeded 70 mW. Imaging was carried out at a depth of 150–300 µm below the surface of the brain.

### Visual stimuli

Visual stimuli were generated using the python library PsychoPy^66^ running on Linux and displayed on two LED backlit monitors (BenQ XL2410T, isoluminant at 25 cd/m^2^, 120 Hz refresh rate, gamma corrected) each positioned 14.5 cm from the eyes and a 45° angle from the longitudinal axis of the animal. For all experiments, stimuli were sinusoidal gratings drifting in the direction that cells were most responsive to (80% contrast, 10 s duration). The size of the stimulus was set at 20° of visual field, and spatial and temporal frequencies were fixed at 0.04 cpd and 1 Hz respectively. These stimulus parameters have been shown to engage all interneuron types in V1^18^. There was no significant dependence of AI on the distance between the center of the stimulus and the center of a neurons receptive field (Fig. S3), so the stimulus location was chosen empirically to activate as many neurons in the FOV as possible.

The standard experimental protocol consisted of ten presentations of the stimulus with a 10 s interstimulus interval consisting of a uniform grey screen of the same mean luminance. When testing an optogenetic manipulation 20 presentations of the stimulus were made, every second being paired with illumination through an amber LED.

### Monitoring of locomotion

Mice were head-restrained but free to run on a spherical air-supported treadmill^67^. The speed of locomotion was measured with an optical mouse (GT650, gaming mouse) positioned in front of the treadmill. Signals from the mouse were conditioned through an Arduino and digitized in parallel with the signals from the detectors of the microscope. For each experiment, post-hoc analysis of the locomotion trace from the optical reader allowed us to binarize it into “still” and “moving”. First, the trace was smoothed and the baseline calculated as the mode of all the bins, which corresponded to periods of rest. The trace was then averaged over bin periods of 2.5 s. A bin was classified as “moving” if its value was greater than a threshold of three standard deviations above the baseline. To compare visual responses in the “resting” and “moving” states we only used stimulus epochs in which the mouse was continuously in that state and for at least three trials per session. (i.e., responses were not used for analysis if the mouse started or stopped moving during the trial). An example of how these criteria were applied is shown in Fig. 7A. The method of housing mice (described above) encouraged motor activity and they were capable of running consistently during our imaging sessions.

### Analysis of two-photon calcium imaging records

Raw movies acquired as TIFF files were registered and segmented into regions of interest (ROIs) using the Suite-2P package running in MATLAB 2015b and then analyzed further using custom written code in Igor Pro 8 (Wavemetrics) including the analysis package SARFIA^68^. For each cell, the average fluorescence signal within the ROI, F, was background-corrected by averaging the signal in a “halo” of pixels extending ~1.5 times the width of the ROI, excluding any that fell within another ROI. The relative intensity of this background signal was then reduced by a “contamination ratio” equivalent to the space occupied by the ROI itself, estimated at 0.5 here and elsewhere^69,70^, after which it was subtracted from the raw signal. Activity traces were expressed as relative changes in fluorescence by dividing the change in fluorescence at each time point by the baseline fluorescence (Δ*F*/*F*_0_). The baseline *F*_0_ was usually computed as the mode of the entire activity trace when this was close to the minimum of that trace.

For each cell, the Pearson’s correlation coefficients was computed between its activity trace (Δ*F*/*F*_0_) and a stimulus trace binarized as either stimulus off (0) or stimulus on (1) using the StatsLinearCorrelationTest Function in Igor. The threshold for significant positive or negative correlation was set at *p* < 0.05. Measurements of adaptive properties were confined to cells which were significantly positively correlated with the stimulus.

The AI was calculated for each 10 s stimulus trial as the normalized ratio between the average responses during the first two cycles (with a delay of 0.5 s from the onset of the stimulus) and the last two cycles. Measurements were rejected if there was no significant response during the trial. Therefore, cells for which optogenetic manipulation supressed responses completely were excluded from analysis. The average AI for each cell under any given condition (with or without locomotion or an optogenetic manipulation) was calculated as the average across multiple stimulus trials, usually 5–10. Changes in the initial gain were calculated from the average response during the first two cycles of the 1 Hz stimulus.

### Spike rate inference from two-photon calcium imaging records

We used the MLspike algorithm^25^, a Bayesian inference approach based on a realistic biophysical model of calcium dynamics in a neuron and activation of the reporter. MLspike was reported to provide the best estimates of spikes measured electrophysiologically in a benchmarking comparison of ten softwares^26^. The steps in the use of MLSpike are described by the example in Fig. S6. The key parameters used to model the measured GCaMP signal are the Hill coefficient with which binding of Ca^2+^ ions activate the reporter (Fig. S7B), the amplitude of the signal generated by a single spike (Fig. S7D) and its relation to the saturating signal and the time-constant with which the GCaMP signal decays after a single spike (Fig. S7C). These parameters were estimated from recordings lasting at least 100–200 s containing 10–20 epidodes of stimulation, each lasting 10 s. None of these parameters demonstrated a significant correlation with the adaptive properties of the PC in which they were estimated. The fit of the model to the raw GCaMP signal (Fig. S6A) is noiseless because the MLSpike algorithm also estimates and subtracts baseline fluctuations.

### Signal-to-noise ratio and detectability

To assess how neurons of different AI contributed to signalling increases and decreases in contrast we applied signal detection theory^34^ and calculated the sensitivity index *d*′ at each time point during a stimulus of varying contrast as the square root of the SNR^35^

$${{{{{{\boldsymbol{d}}}}}}}^{{\prime} }=\frac{{{{{{{\rm{|}}}}}}{{{{{\boldsymbol{\mu }}}}}}}_{{{{{{\boldsymbol{s}}}}}}}-{{{{{{\boldsymbol{\mu }}}}}}}_{{{{{{\boldsymbol{N}}}}}}}{{{{{\rm{|}}}}}}}{\sqrt{{{{{{{\boldsymbol{\sigma }}}}}}}_{{{{{{\boldsymbol{N}}}}}}}^{{{{{{\bf{2}}}}}}}}},$$

where *μ*_s_ is the mean amplitude of the signal over a given 2 s window and *μ*_N_ is the mean signal over a 2 s window immediately preceding it, while the noise *σ*^2^_N_ is the standard deviation over the same preceding window. In other words, *σ*^2^_N_ was not measured across trials but within trials. We then averaged this running measure of *d*′ in each cell from multiple trials (usually five).

### Optogenetics

Red-shifted optogenetic activators (ChrimsonR or ArchT) were excited through the objective using an amber LED (Thorlabs, 590 nm, M590l3) controlled through a high-power LED driver (DC2200). To prevent contamination of GCaMP signals, the LED was pulsed to deliver light only during the turning phase of the x-mirror of the microscope, as monitored through the position signal of the mirror controller (Cambridge Technology). An Arduino read the position signal from the mirror controller (Cambridge Technology) and delivered a TTL signal for the LED driver. Under our usual imaging conditions, the LED power was pulsed at 2 KHz, each pulse lasting ~0.1 ms.

Where the effect of the optogenetic manipulation was to inhibit PCs, it was important not to completely suppress the initial response so as to quantify a change in AI. LED power was therefore calibrated during each imaging session to ensure that the average initial response of a population of PCs within a FOV was not reduced by more than 75% or by less than 25% (Fig. S8A, B, average reduction across all selected FOVs was 53 ± 16%). This was achieved using powers of 8–60 µW out of the objective when using ChrimsonR (equivalent to peak intensities of 20–150 µW mm^−2^ at the focal plane).

Where the effect of the optogenetic manipulation was to increase PC activity, LED power was adjusted to ensure that the initial response of most PCs was increased by at least 50% but not more than ~200% (Fig. S8C, D, average increase 144 ± 73%). This was achieved using powers of 0.6–4.9 mW using ArchT. An increase of 300% was deemed within the physiological range because it was often observed when the animal transitioned from rest to locomotion. When PC responses were increased by over-activating VIP interneurons using ChrimsonR the LED power ranged from 60 µW to 4.9 mW.

### Statistics

To test if the mean of the distribution of AIs in a given condition differed significantly from zero we used both *t*-tests and a non-parameteric approach in which we created a sampling distribution by bootstrapping with replacement (half the number of samples of the original, 5000 repeats). The use of a *t*-test is validated by the Gaussian distribution of AIs (Fig. 1G). There was no difference to the inferences that we could make using either test and both are reported where appropriate.

To test if the mean of the distribution of AIs in a given condition differed significantly from another condition we usually made measurements on a *paired basis*. This includes the effects of a transition from rest to locomotion (Fig. 8E) and various optogenetic manipulations, where we specifically used an experimental protocol which interleaved stimuli with and without the manipulation so that we could then use of statistical tests for paired variables (Figs. 4–6). In these situations we could not use a bootstrapping approach because the variables are not independent (each being measured within the same cell while AI varies from cell-to-cell). We therefore used a non-parametric Wilcoxon Signed Rank test for paired variables as implemented by the StatsWilcoxonRankTest function in IgorPro, where the null hypothesis is that the data in the two conditions are statistically the same.

### Reporting summary

Further information on research design is available in the Nature Research Reporting Summary linked to this article.

## Supplementary information

Supplementary Information

Reporting Summary

## Data Availability

All the data supporting the results of this investigation are present within the article, supplementary file or source data provided with the paper. Further information and requests for resources and reagents are available upon request from the corresponding author. Enquiries should be directed to Leon Lagnado (L.Lagnado@sussex.ac.uk). Source data are provided with this paper.

## Source data

Source Data

## Code availability

Custom code used for data analysis is available upon request to Leon Lagnado (L.Lagnado@sussex.ac.uk).
