# Publisher Correction: Opposite forms of adaptation in mouse visual cortex are controlled by distinct inhibitory microcircuits

**DOI:** 10.1038/s41467-022-29509-9

**Published:** 2022-03-30

**Authors:** Tristan G. Heintz, Antonio J. Hinojosa, Sina E. Dominiak, Leon Lagnado

**Affiliations:** Sussex Neuroscience, School of Life Sciences, University of Sussex, Brighton, BN1 9QG UK

**Keywords:** Neuroscience, Sensory processing

Correction to: *Nature Communications* 10.1038/s41467-022-28635-8, published online 24 February 2022.

The original version of this Article contained an error in Fig. 5. The titles for Fig. 5a and Fig. 5g were incorrectly given as “SST ChrimsonR (gain ")” and “SST ChrimsonR (gain !)” instead of “SST ChrimsonR (gain ↓)” and “SST ChrimsonR (gain ↑)”, respectively. This has been corrected in the PDF and HTML versions of the Article.

