# Supplementary material for: Opposite forms of adaptation in mouse visual cortex are controlled by distinct inhibitory microcircuits
Source: Nat Commun. 2022 Feb 24;13:1031. doi: 10.1038/s41467-022-28635-8 (PMC8873261; doi:10.1038/s41467-022-28635-8)
Supplement: Supplementary file 1 — Supplementary Information [file 41467_2022_28635_MOESM1_ESM.pdf]

Supplementary Information

Opposite forms of adaptation in mouse visual cortex are controlled by distinct inhibitory microcircuits

Figure S1: Cross-validation of metrics

Figure S2: Comparison of the distribution of adaptive indices across fields of view and between mice.

Figure S3. The adaptative index did not depend on stimulus location relative to receptive field.

Figure S4. Orientation/direction preference had no influence on the Adaptive Index of pyramidal neurons

Figure S5. Comparisons of the decay kinetics of the GCaMP6f signal.

Figure S6. Estimating spiking activity from GCaMP signals using MLSpike.

Figure S7. Variations in Adaptive Index estimated from GCaMP signals could not be accounted for by variations in the physiological properties of pyramidal cells.

Figure S8. Selection of appropriate LED intensities to photoactivate ChrimsonR and ArchT

Figure S9. Changes in Adaptive Index of PCs caused by optogenetic activation of interneurons.

Figure S10. Optogenetic manipulations did not have long-term effects on PC activity.

Figure S11. Optogenetic activation and inhibition of SST interneurons.

Figure S1

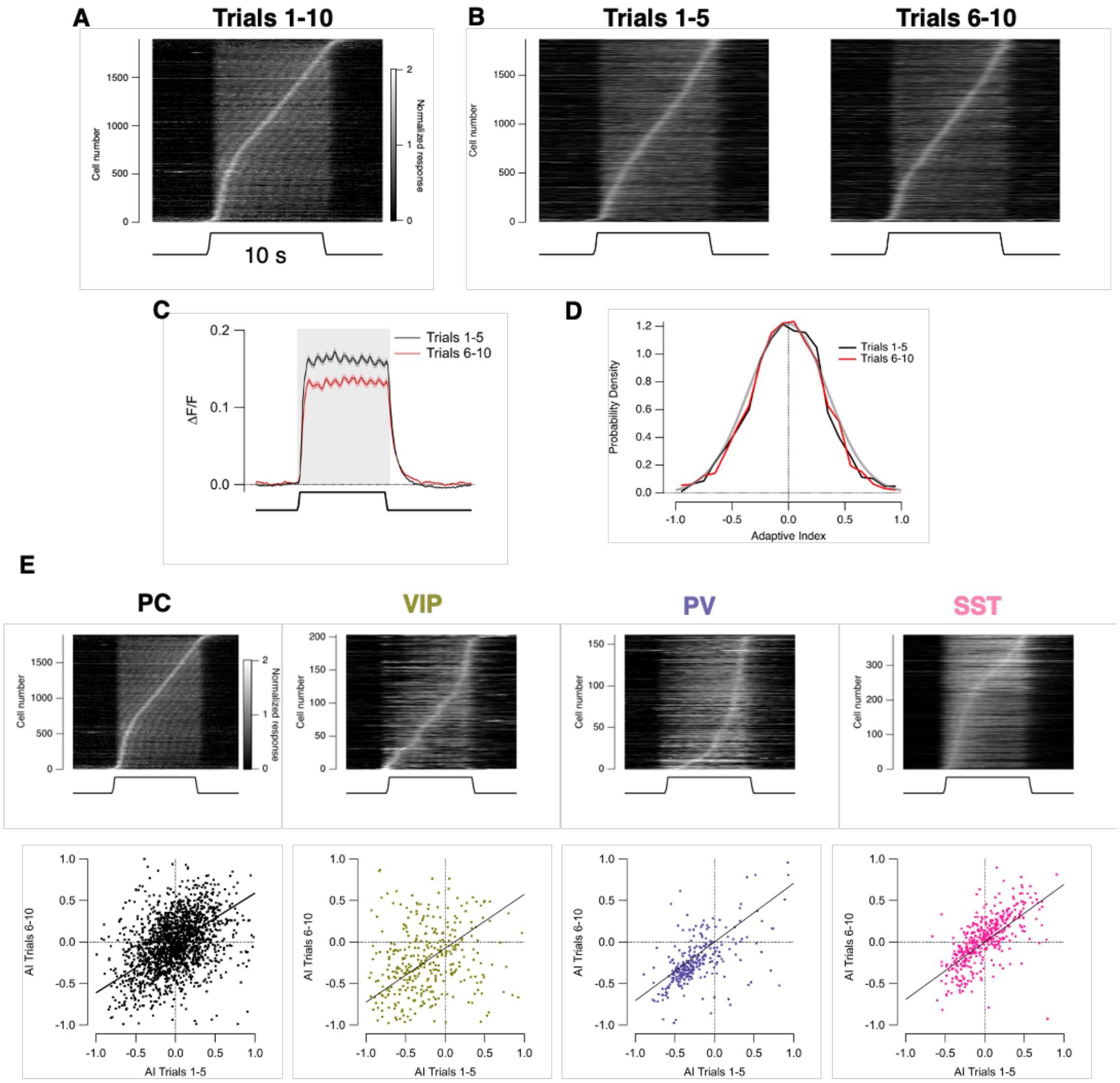

Figure S1. Cross-validation of metrics

A. Raster plot showing responses of 1866 PCs to a high-contrast stimulus applied for 10 s (average of 10 trials, collected from 18 mice). Responses have been normalized and sorted according to time of peak (as shown in Fig. 1C). **B.** Separate raster plots for the first 5 trials (left) and second five (right) using the same ordering of cells as in A. **C.** Comparison of the average response of PCs in the first and second set of trials. The response fell by ~15% in later trials. **D.** Comparison of the distribution of Adaptive Indices in the first and second set of trials (no significant difference by Wilcoxon Signed Rank test for paired variables, $p = 10^{-5}$). The Gaussian fit to the average of the two distributions is centred on AI = -0.005 with and sd of 0.35. **E.** Comparison of raster plots (top) in PCs, VIP, PV and SST interneurons (as in A). PV and VIP cells are predominantly sensitizing (peaking towards the end of the 10 s stimulus) while SST cells are predominantly depressing (peaking early). The lower graphs show scatter plots of the distribution of AI in the second set of five trials compared to the first set on a cell-by-cell basis. The slopes of the lines through the origin are: PCs, 0.6; VIP cells, 0.65; PV cells, 0.71, and SST cells, 0.69. In all cases the average AI in the first set of trials was significantly correlated with that in the second set (Spearman's Rank Correlation test p = 0.01). Data are represented as mean ± SEM (grey and red shading area in c) or probability density (d). Source data are provided as a Source Data file.

Figure S2

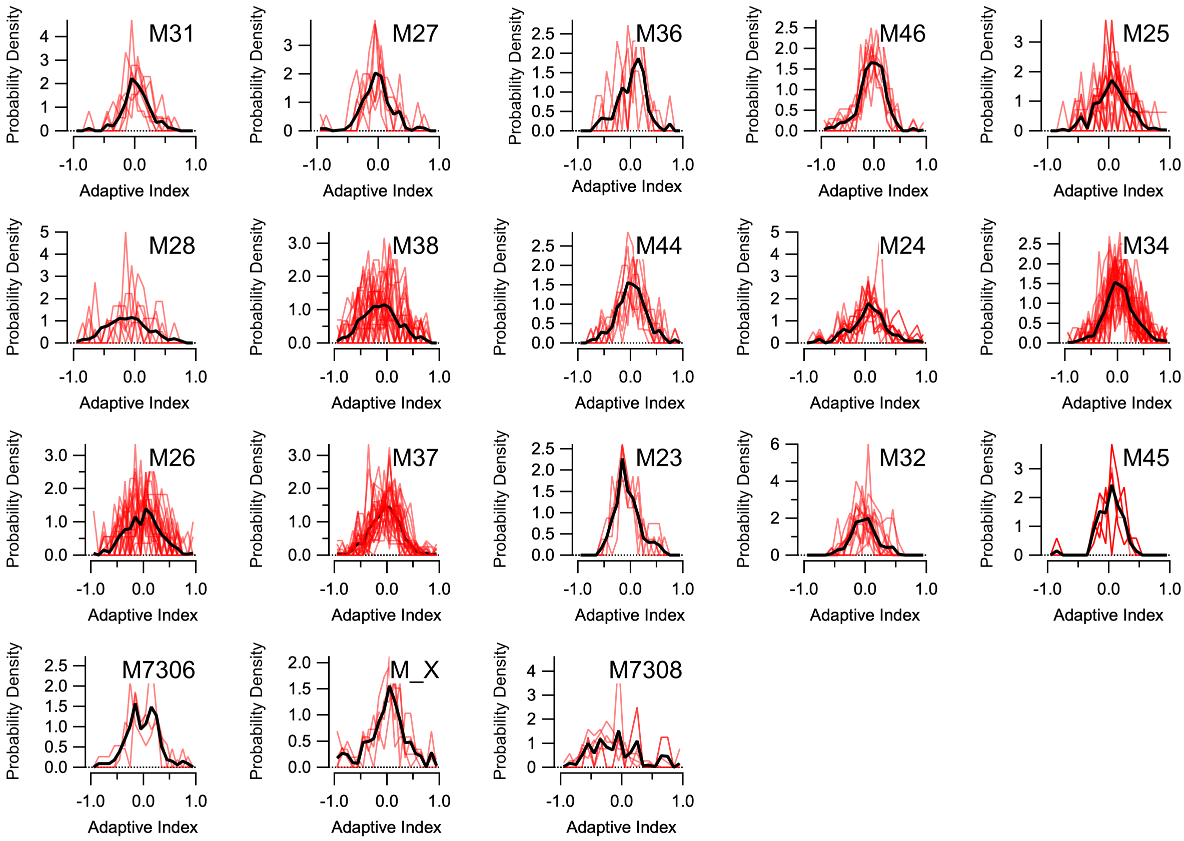

Figure S2. Comparison of the distribution of adaptive indices across fields of view and between mice. Each graph represents one mouse. Each red trace represents the distribution of AIs in one field of view in one recording session and the bold black trace is the average of these. These distributions can be compared with the average across all 18 mice shown in Fig.1G. An approximate balance between depressing and sensitizing adaptation was a consistent feature of V1. Source data are provided as a Source Data file.

Figure S3

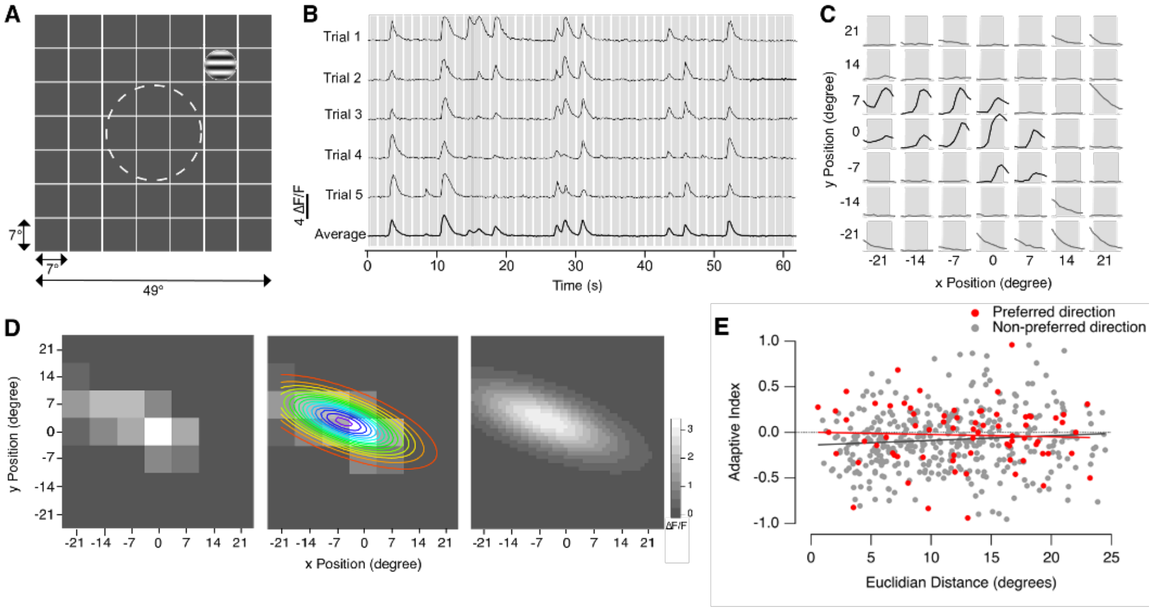

Figure S3. The adaptative index did not depend on stimulus location relative to receptive field. These experiments began with the standard adapting stimulus (20° diameter) presented in the center of the visual field 10 times to determine the adaptive index of each neuron in the same field of view.

A. Schematic of the stimulus protocol used to estimate the center of each neurons receptive field. A circular drifting grating (7° diameter, 80% contrast, 0.04 cpd, 1 Hz, moving upwards) was presented at 49 locations for 1 s. The sequence of stimuli was chosen so that consecutive stimuli were at least two squares apart. All locations were stimulated 5 times. **B.** An example of the responses of a PC to the 49 stimulus locations. The bold trace at the bottom shows the average response to the 5 trials. Grey bars represent the timing of the stimulus at each of 49 locations. The neuron responded reliably to a subset of stimulus locations. **C.** The average response of the same neuron shown at the location of the stimulus. **D.** Heat map (left) that represents the peak of the response at a certain stimulus location. Note that only upward deflections in C were counted as a response. A monotonic fall in the GCaMP signal represents recovery from a stimulus eliciting a response at the previous location. A 2D Gaussian fit was applied to the heat map (middle and right) from which the receptive field center was estimated. **E.** Scatter plot showing the AI of each cell as a function of the Euclidian distance between the center of its receptive field (estimated as in A-D) and the center of the adapting stimulus. The line fitted to the points at the non-preferred direction (black) has a slope of 0.005 ± 0.003 and the correlation was significant at p = 0.05 (Spearman's rank correlation test). The AI therefore had a weak tendency towards sensitization the closer the stimulus was to the center of the PCs receptive field. Results from 451 cells from 4 mice. The line fitted to the points when the adapting stimulus was at the preferred direction (red) has a slope of -0.003 ± 0.007 and the correlation was not significant. Source data are provided as a Source Data file.

Figure S4

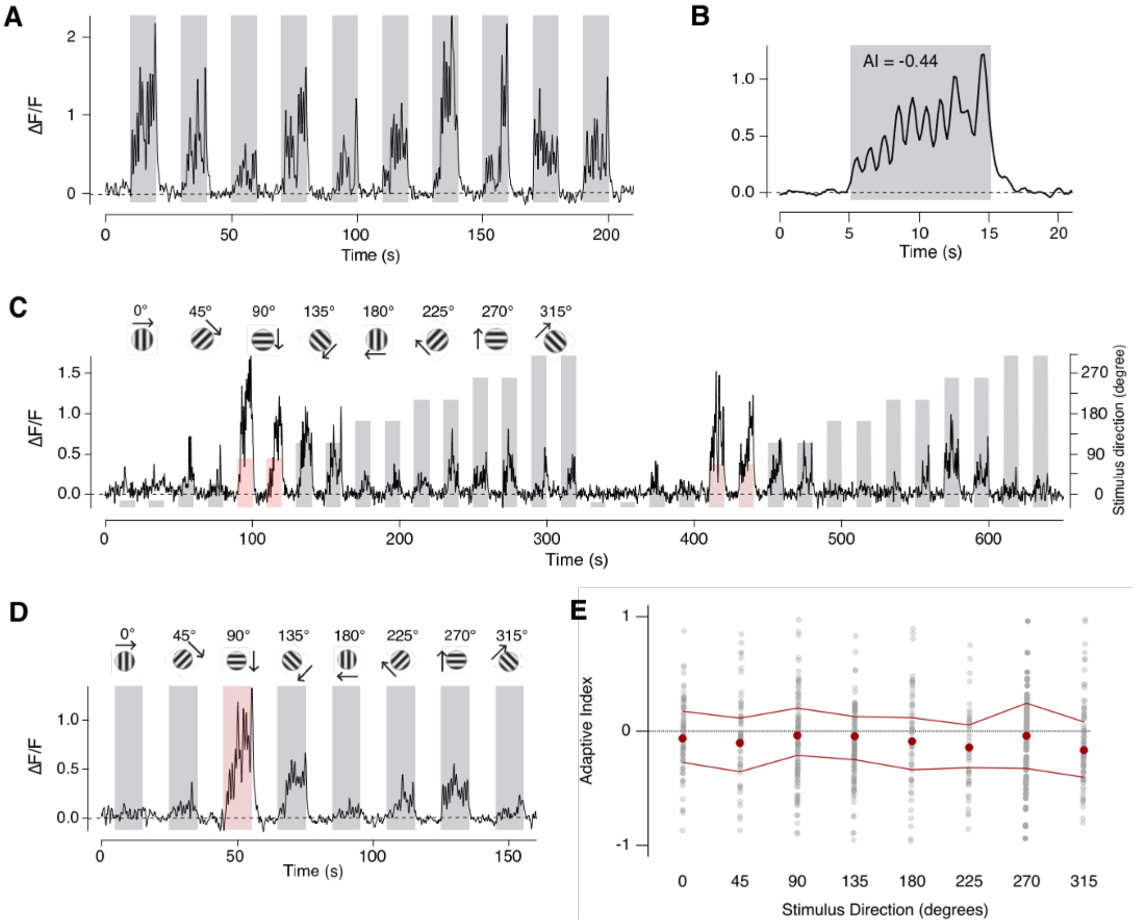

Fig. S4. Orientation/direction preference had no influence on the Adaptive Index of pyramidal neurons

A-C show the experimental approach to testing effects of stimulus orientation and direction. **A.** First, the Adaptive Index of each neuron was measured using the standard protocol of 10 stimulus trials in which the grating was moved at 270º (horizontal bars moving upwards). An example response from one PC. The time of each stimulus is indicated by grey bars. **B.** The average response of the neuron shown in A (AI= -0.44). **C.** The neuron was then tested with a grating drifting in eight different grating directions (between 0° – 315°) and four different orientations. All directions were presented twice in each trial and the animal was exposed to two trials overall. The different heights of the stimulus bars represent different directions. The pink bars indicate the direction at which the neuron was most responsive i.e the preferred direction. **D.** Average response of the same neuron to all 8 directions. The preferred direction (in this case 90°, pink bar) was identified as the highest average response amplitude over the course of the stimulus duration. **E.** Scatter plot showing the AI of each neuron as a function of stimulus direction relative to preferred direction (zero degrees represents the preferred direction). Red circles are the average AI of all cells and red lines show upper and lower quartiles. The AI of PCs was independent of the stimulus direction as judged by a nonparametric multiple comparison test based on the Dunn-Hollander-Wolfe approach comparing each direction to the control at 270º (implemented using the StatsNPMCTest function in IgorPro). Results from 675 PCs imaged from 4 mice. Source data are provided as a Source Data file.

Figure S5

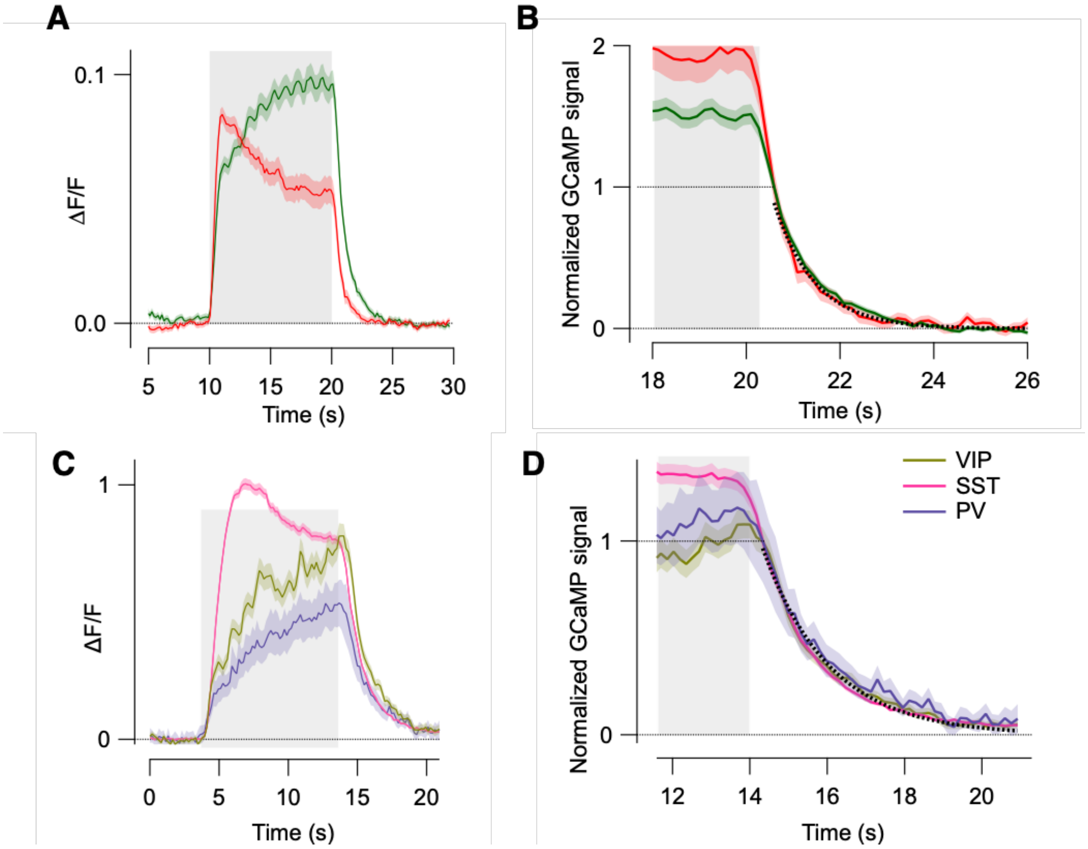

Figure S5. Comparisons of the decay kinetics of the GCaMP6f signal.

A. Averaged response from the 25% of PCs with lowest AI (green, sensitizing) and the 25% with the highest AI (red, depressing). Results are from the distribution in Fig. 1G (1896 cells, 18 mice). **B.** The falling phases of the GCaMP6f signal after normalization to the signal 0.25 s after the end of the stimulus. The dashed line is an exponential fit to the red trace constrained to decay to zero, which provides an estimate of 0.87 ± 0.03 s for the decay time-constant (mean ± sd).. The kinetics of decay of the calcium signal were similar in sensitizing and depressing PCs, indicating that the different kinetics of the calcium signal during the stimulus were unlikely to be due to variations in the rate of calcium extrusion. The average value of tau calculated in this way was larger than that estimated from the estimate of 0.66 s from decay of smaller transients in Fig. S7C, likely because spiking did not cease immediately after the end of the stimulus. **C.** Averaged responses of VIP, SST and PV interneurons to the high-contrast stimulus are shown superimposed (from Fig. 3A; stimulus shown by grey bar). **D.** The falling phases of the responses after normalization to the signal 0.26 s after the end of the stimulus. The dashed line is an exponential fit decaying with a time-constant of 1.73 s. The kinetics of decay of the calcium signal were not significantly different in sensitizing (VIP ad PV) and depressing (SST) interneurons, indicating that the different kinetics of the calcium signal during the stimulus were unlikely to be due to variations in the rate of calcium extrusion. Data are represented as mean ± SEM (coloured shading areas). Source data are provided as a Source Data file.

Figure S6

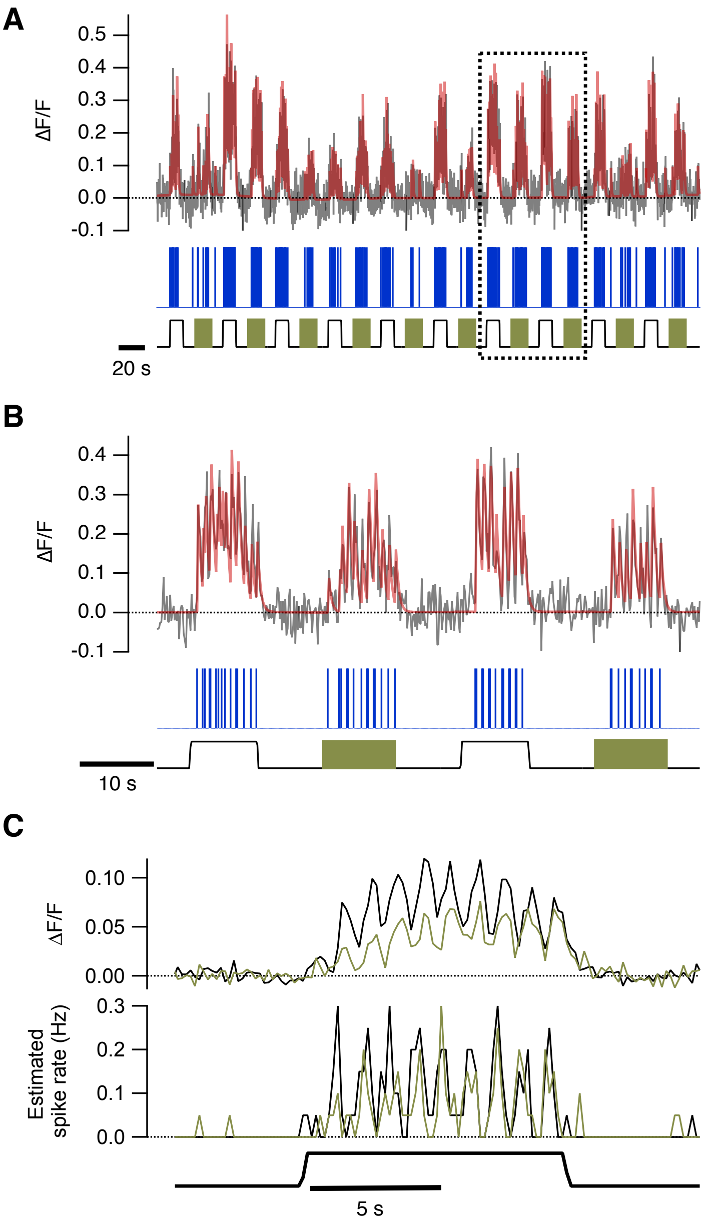

Figure S6. Estimating spiking activity from GCaMP signals using MLSpike.

A. Example of the GCaMP signal from a PC (grey) together with the most likely spike train (blue) and the calcium signal reconstructed from the train (red). In this experiment a high-contrast stimulus was applied in 10 s trials, as shown by the bottom trace. The 10 control trials were interleaved with 10 trials in which VIP interneurons were inhibited optogenetically using ArchT (green). **B.** Expansion of the period shown in the black dashed box in A. Note the similarity between the GCaMP signal (grey) and the fit (red) provided by the physiological model that relates the most likely spike train to a GCaMP signal. The fit to the raw GCaMP signal is noiseless because the MLSpike algorithm estimates and subtracts baseline fluctuations. **C.** Top: the GCaMP signal averaged over 10 control trials (black) compared with the signal generated during inhibition of VIP interneurons (green). Bottom: the same comparison for the reconstructed spike trains. Other examples of the relation between GCaMP signal and estimated spike train for individual neurons are shown in Fig. 1D-F. Collected results showing the effects of inhibiting VIP interneurons on the gain and adaptive properties of PCs are shown in Fig. 6D-F. Source data are provided as a Source Data file.

Figure S7

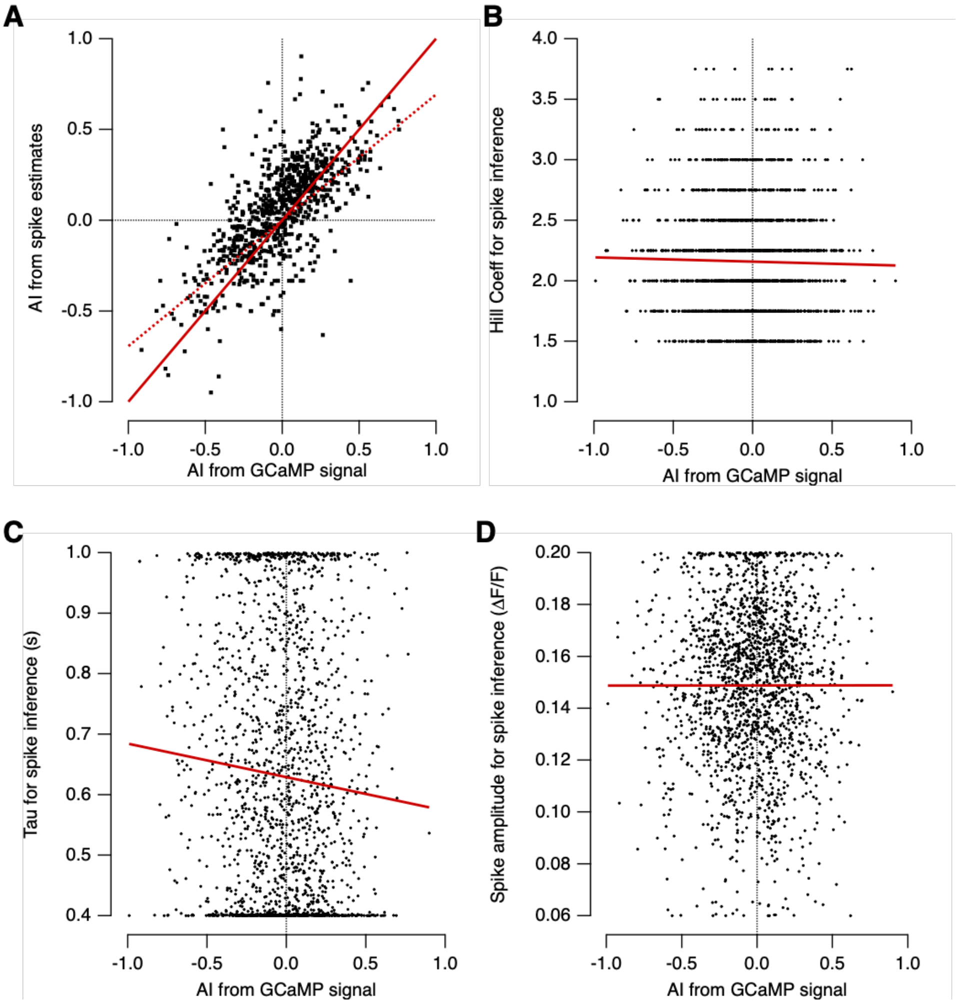

Figure S7. Variations in Adaptive Index estimated from GCaMP signals could not be accounted for by variations in the physiological properties of pyramidal cells.

A. The relation between the AI calculated from the inferred spike train and the raw GCaMP signal. The red line represents unity and the dashed line is a best fit constrained to pass through the origin with slope 0.70 ± 0.03. (the correlation coefficient). Each point represents one of the 1896 cells with spike responses estimated as shown in Fig. 1B and C. **B.** The relation between the Hill coefficient (H) for calcium binding to GCaMP6f and AI calculated from the GCaMP signal. H was estimated by MLSpike to provide the best description of the GCaMP signal and averaged 2.16 ± 0.48. The correlation coefficient was not significantly different from zero (r = -0.022 ± 0.023, mean ± sem). Values of H between 1.5 and 4 were tested in steps of 0.25. **C.** The relation between the time-constant for the decline in the GCaMP signal (tau) and the AI calculated from the GCaMP signal. Tau averaged 0.63 ± 0.01 s and the red line has a slope of -0.06 ± 0.02 and limits of 0.66 s at AI = -1 and 0.58 s at AI =1. For likelihood estimation of spikes, values of tau were therefore constrained between 0.4 s and 1 s. **D.** The relation between the amplitude of the GCaMP signal generated by a single spike and the calculated AI. The spike amplitude averaged a ΔF/F value of 0.15 ± 0.001 and the red line fitted to the data has a slope of 0.011 ± 0.003 with limits of 0.1487 at the lowest AI measured (-0.99) and 0.1488 at the highest (0.90). For likelihood estimation of spikes, values of spike amplitude were constrained between 0.06 s and 0.2. Source data are provided as a Source Data file.

Figure S8

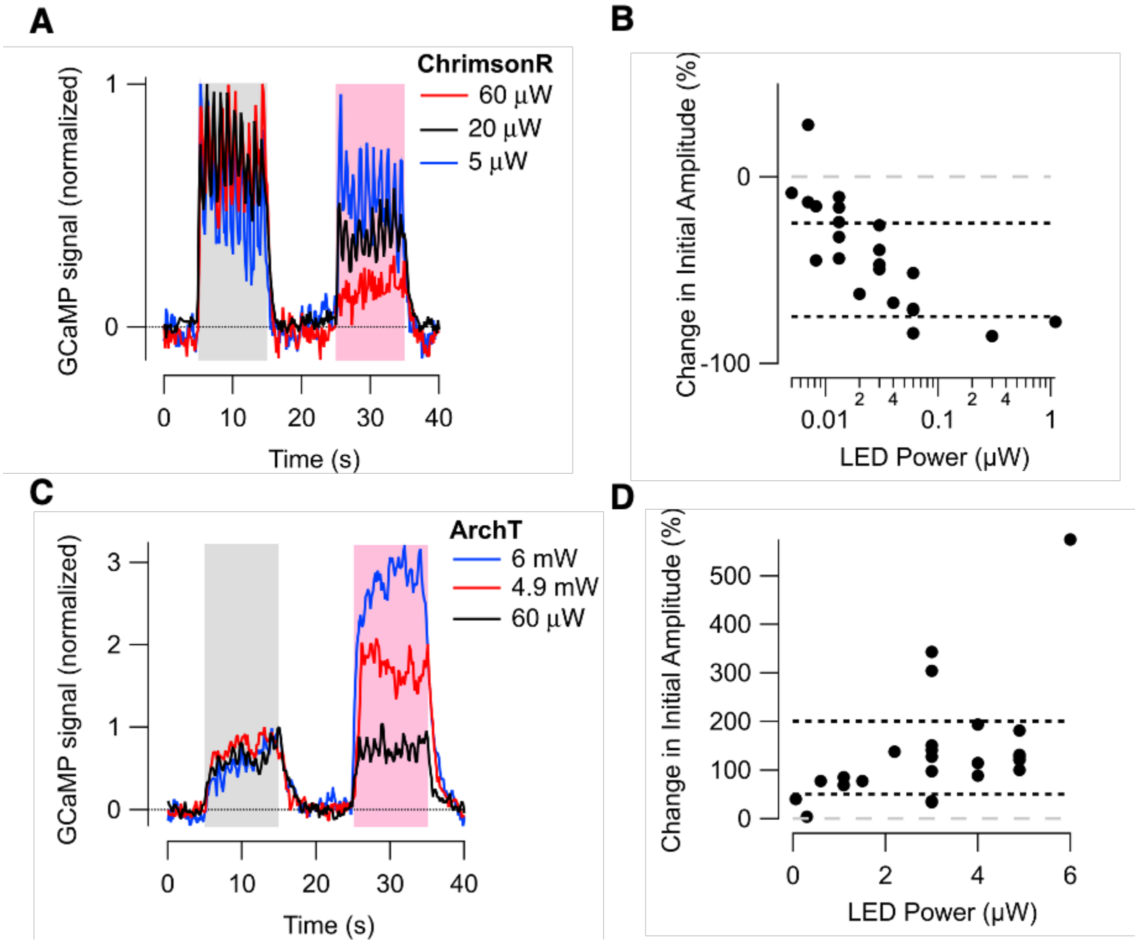

Figure S8. Selection of appropriate LED intensities to photoactivate ChrimsonR and ArchT

A. Average activity of PCs in one FOVs in respons to the standard high-contrast stimulus applied in control conditions (grey bar) and during photostimulation of SST interneurons expressing Chrimson R (pink bar). LED intensities were 60 µW (red), 20 µW (black) and 5 µW (blue). The intermediate intensity caused the initial amplitude of the response to decline by ~50% and was therefore chosen as appropriate for this experiment (see Methods). **B.** Scatter plot showing the distribution of the relative change in initial amplitude when stimulating ChrimsonR in SST interneurons. Each point is a single FOV. LED intensities were chosen to reduce initial amplitude by 25-75% (black dashed lines) which as achieved at LED intensities of 8 - 60 µW. Grey line shows no change. Similar ranges were found for ChrimsonR in PV interneurons. **C.** Same as A but for activation of ArchT in SST interneurons. LED intensities were 6 mW (blue), 4.9 mW (red) and 60 µW (black). The intermediate intensity caused the initial amplitude of the response to increase by ~150% and was therefore chosen as appropriate for this experiment. **D.** Scatter plot showing the distribution of the relative change in initial amplitude when stimulating ArchT in SST interneurons. Each point is a single FOV. LED intensities were chosen to increase initial amplitude by 50-200% (black dashed lines) which as achieved at LED intensities of 0.6 - 4.9 mW. Grey line shows no change. Data are represented as mean ± SEM (coloured shading area in a, c). Source data are provided as a Source Data file.

Figure S9

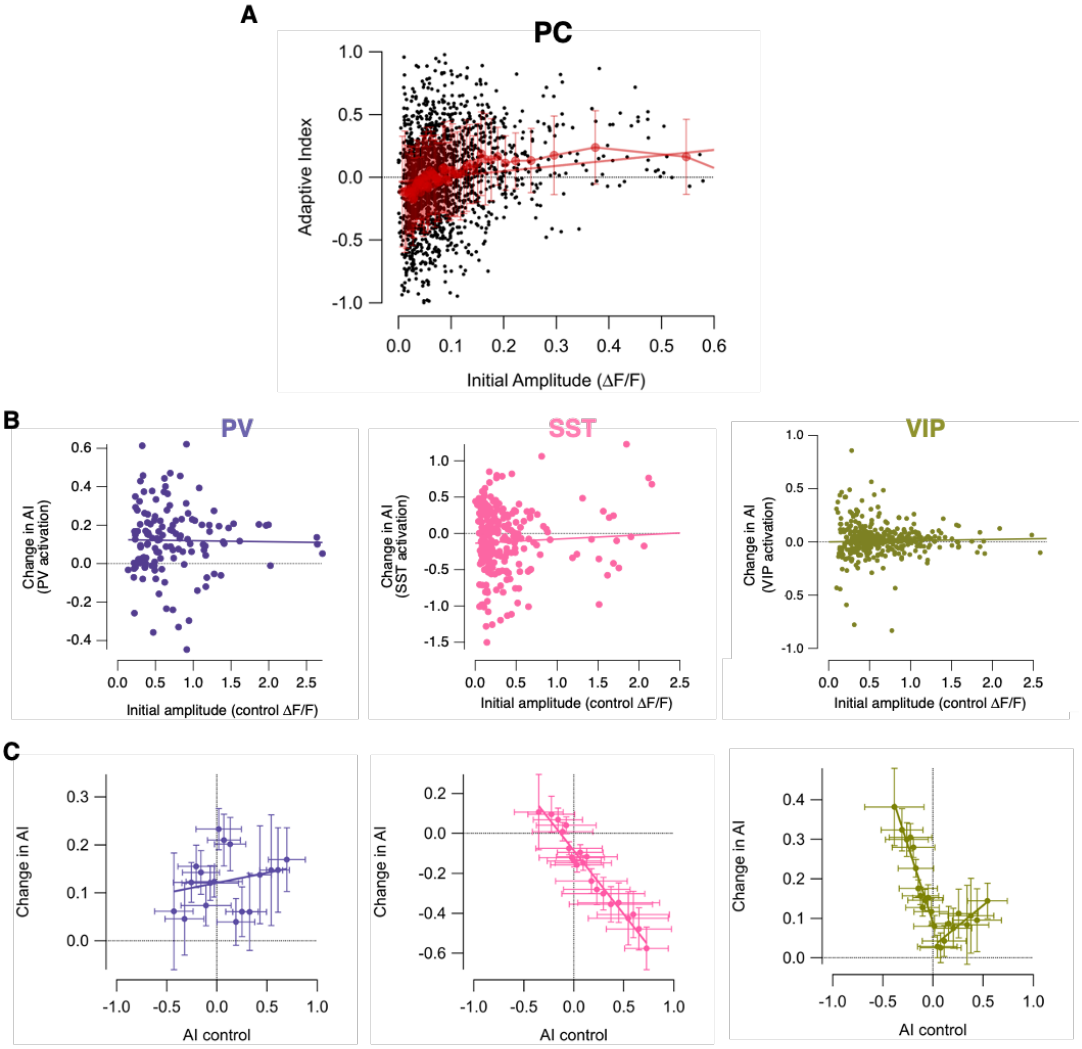

Figure S9. Changes in Adaptive Index of PCs caused by optogenetic activation of interneurons.

A. The change in AI in PCs as a function of the initial amplitude of the response. The black dots show individual cells (1754 from 18 mice) and the red circles averages of groups of 30 in ascending order of AI. . The slope of the line fitted to the points was not significantly different from zero (0.42 ± 0.33, mean ± sd). **B.** The change in AI in PCs caused by over-activation of interneurons expressing ChrimsonR is plotted as a function of the initial amplitude of the response measured under control conditions. Each point represents one neuron (PV, 296 cells from 3 mice; SST, 401 cells from 3 mice; VIP, 414 cells from 3 mice). The line fitted to the points have slopes of: PV, -0.006 ± 0.032 (mean ± sd); SST, 0.06 ± 0.07, and VIP, 0.01 ± 0.01. None are significantly different from zero (WSR test): there is therefore no correlation between the direction of the adaptive change and the amplitude of the initial response. **C.** The change in AI in PCs caused by over-activation of interneurons expressing ChrimsonR is plotted as a function of the AI under control conditions. For clarity, paired measurements were binned. PV cell activation showed no significant correlation (slope of fitted line 0.04 ± 0.04). SST cells showed a negative correlation, indicating a shift towards sensitization for all starting AI (slope = -0.64 ± 0.03). Overactivation of VIP interneurons caused a shift towards depression where the control AI was less than zero (slope =- 0.78 ± 0.53) and when control AI was more than zero (0.21 ± 0.42). Data is expressed as mean ± SEM. Source data are provided as a Source Data file.

Figure S10

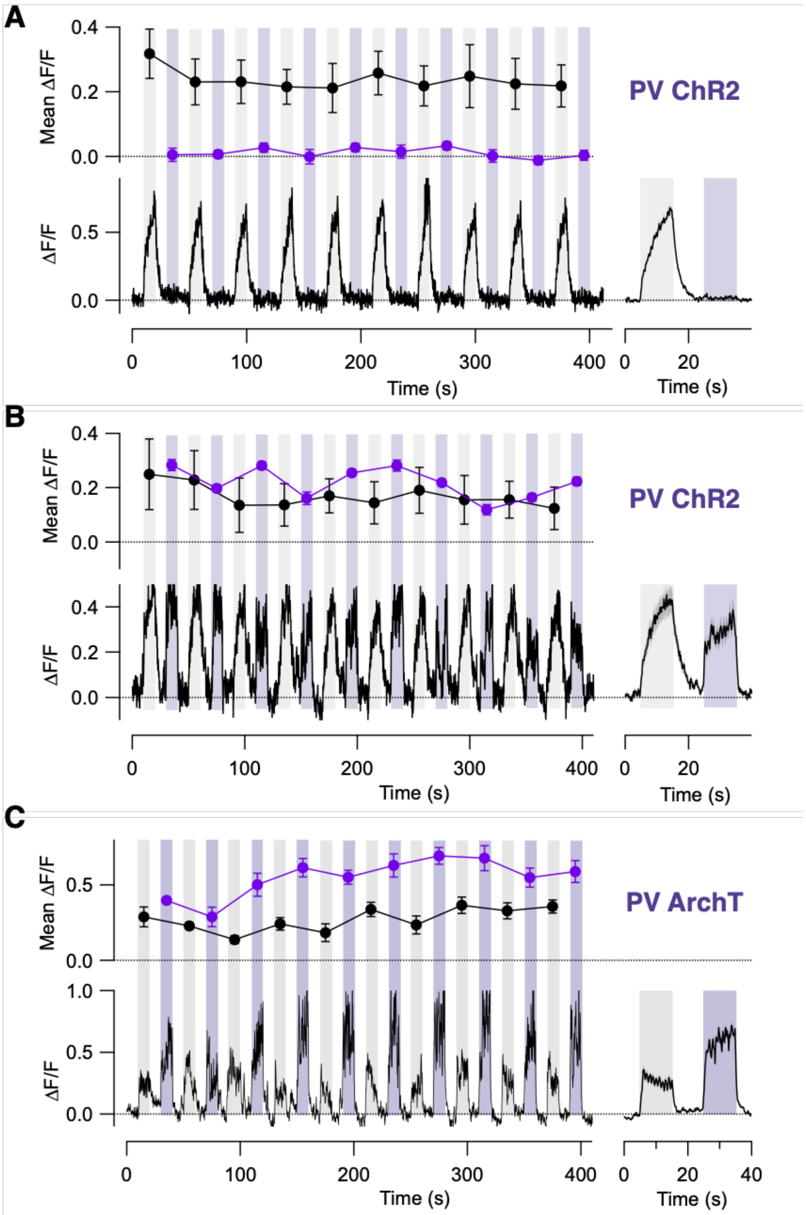

Figure S10. Optogenetic manipulations did not have long-term effects on PC activity.

A. GCaMP6f activity in a PC in which activating PV interneurons using ChrimsonR blocked visual responses. The lower trace is averaged from 10 repetitions of the protocol Stimulus trials with LED illumination (purple bars) and without (grey bars) were interleaved. To the right is the average response under the two conditions from the same series of trials. Above the GCaMP trace is a plot of the average response in each trial. Note that control trials (black) were relatively stable over the course of the experiment. To the right is shown the average amplitude of the responses in control conditions (grey bar) and during optogenetic activation of PV interneurons (purple bar). **B.** A second example where overactivating PV interneurons had a weaker effect (average responses from 18 PCs in one FOV - the same as featured in Fig. 4D). AI = -0.28 ± 0.03 in control conditions and -0.05 ± 0.03 during PV activation. **C.** Average responses from 43 PCs in one FOV (as featured in Fig. 4G). AI = 0.04 ± 0.05 in control conditions and -0.09 ± 0.05 during PV inhibition. The optogenetic manipulation was to inhibit PV interneurons expressing ArchT. AI = 0.04 ± 0.05 in control conditions and -0.09 ± 0.05 during PV inhibition. Data are represented as mean ± SEM . Source data are provided as a Source Data file.

Figure S11

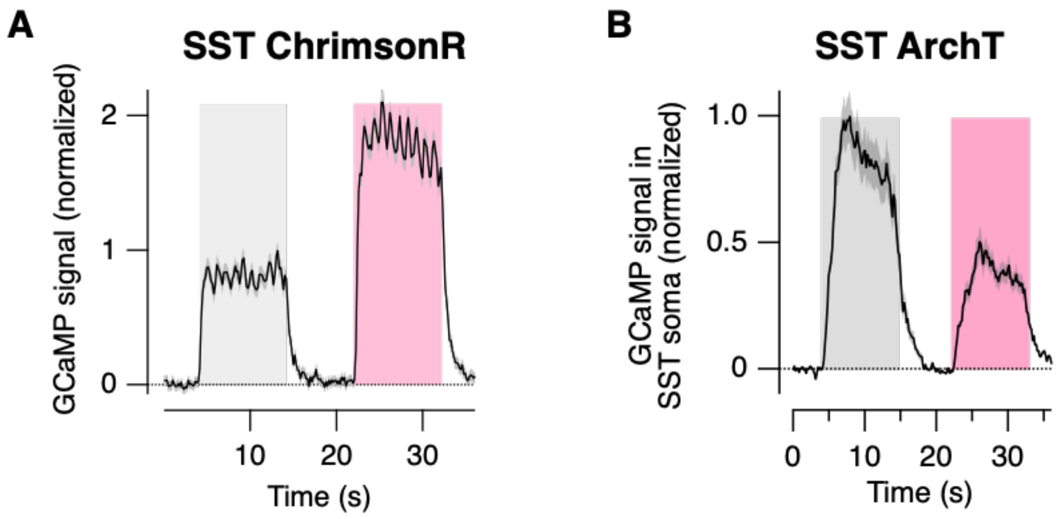

Figure S11. Optogenetic activation and inhibition of SST interneurons.

A. Population average of GCaMP6f response in SST interneurons in response to the high-contrast stimulus delivered without (grey bar) and with (pink bar) optogenetic activation using ChrimsonR (41 cells, 2 mice). The signal has been normalized to the maximum under control conditions. The activity of SST cells approximately doubled. Note that these averages were calculated from interleaved trials, as shown in Fig. 5, and therefore indicate that the average level of SST neuron activation during the protocol used to investigate the effect on PC activity. **B.** As in A, except that the optogenetic manipulation was to inhibit SST interneurons using ArchT (40 cells, 1 mouse). The activity of SST cells was approximately halved (see also Fig. 5D-F). Data are represented as mean ± SEM (grey shading area). Source data are provided as a Source Data file.
