# Supplementary material for: Opposite forms of adaptation in mouse visual cortex are controlled by distinct inhibitory microcircuits
Source: Nat Commun. 2022 Feb 24;13:1031. doi: 10.1038/s41467-022-28635-8 (PMC8873261; doi:10.1038/s41467-022-28635-8)
Supplement: Supplementary file 2 — Reporting Summary [file 41467_2022_28635_MOESM2_ESM.pdf]

nature research

Corresponding author(s): Leon Lagnado

Last updated by author(s): Jan 23, 2022

Reporting Summary

Nature Research wishes to improve the reproducibility of the work that we publish. This form provides structure for consistency and transparency in reporting. For further information on Nature Research policies, see our Editorial Policies and the Editorial Policy Checklist.

Statistics

For all statistical analyses, confirm that the following items are present in the figure legend, table legend, main text, or Methods section.

n/a	Confirmed	
☐	☒	The exact sample size (n) for each experimental group/condition, given as a discrete number and unit of measurement
☐	☒	A statement on whether measurements were taken from distinct samples or whether the same sample was measured repeatedly
☐	☒	The statistical test(s) used AND whether they are one- or two-sided<br>*Only common tests should be described solely by name; describe more complex techniques in the Methods section.*
☐	☒	A description of all covariates tested
☐	☒	A description of any assumptions or corrections, such as tests of normality and adjustment for multiple comparisons
☐	☒	A full description of the statistical parameters including central tendency (e.g. means) or other basic estimates (e.g. regression coefficient) AND variation (e.g. standard deviation) or associated estimates of uncertainty (e.g. confidence intervals)
☐	☒	For null hypothesis testing, the test statistic (e.g. F, t, r) with confidence intervals, effect sizes, degrees of freedom and P value noted<br>*Give P values as exact values whenever suitable.*
☒	☐	For Bayesian analysis, information on the choice of priors and Markov chain Monte Carlo settings
☒	☐	For hierarchical and complex designs, identification of the appropriate level for tests and full reporting of outcomes
☒	☐	Estimates of effect sizes (e.g. Cohen's d, Pearson's r), indicating how they were calculated

Our web collection on statistics for biologists contains articles on many of the points above.

Software and code

Policy information about availability of computer code

Data collection	Fluorescence was measured with a two-photon microscope (Scientifica SP1, galvonometer mirrors) controlled using Scanimage 5 software (Vidrio Technologies). Visual stimuli were generated using the python library PsychoPy (Peirce, 2007) .
Data analysis	Raw movies acquired as TIFF files were registered and segmented into Regions of Interest (ROIs) using the Suite-2P package running in MATLAB 2015b and then analyzed further using custom written code in Igor Pro 8 (Wavemetrics) including the analysis package SARFIA50.

For manuscripts utilizing custom algorithms or software that are central to the research but not yet described in published literature, software must be made available to editors and reviewers. We strongly encourage code deposition in a community repository (e.g. GitHub). See the Nature Research guidelines for submitting code & software for further information.

Data

Policy information about availability of data

All manuscripts must include a data availability statement. This statement should provide the following information, where applicable:

- Accession codes, unique identifiers, or web links for publicly available datasets
- A list of figures that have associated raw data
- A description of any restrictions on data availability

The datasets generated during and/or analysed during the current study are available from the corresponding author on reasonable request.

Field-specific reporting

Please select the one below that is the best fit for your research. If you are not sure, read the appropriate sections before making your selection.

☒ Life sciences ☐ Behavioural & social sciences ☐ Ecological, evolutionary & environmental sciences

For a reference copy of the document with all sections, see nature.com/documents/nr-reporting-summary-flat.pdf

Life sciences study design

All studies must disclose on these points even when the disclosure is negative.

Sample size	Sample sizes were chosen according to the average effect of an optogenetic manipulation relative to the variability in measurements.
Data exclusions	No data were excluded.
Replication	The effects of optogenetic manipulations were tested in at least 3 mice to ensure replicability.
Randomization	Both contol and test measurements were made as paired measurements in the same populations of neurons.
Blinding	Blinding is not relevant to the study. But we ensured that blind analysis by 2-3 members of the lab produced similar results from a given data set.

Reporting for specific materials, systems and methods

We require information from authors about some types of materials, experimental systems and methods used in many studies. Here, indicate whether each material, system or method listed is relevant to your study. If you are not sure if a list item applies to your research, read the appropriate section before selecting a response.

Materials & experimental systems

n/a	Involved in the study	
☒	☐	Antibodies
☒	☐	Eukaryotic cell lines
☒	☐	Palaeontology and archaeology
☐	☒	Animals and other organisms
☒	☐	Human research participants
☒	☐	Clinical data
☒	☐	Dual use research of concern

Methods

n/a	Involved in the study	
☒	☐	ChIP-seq
☒	☐	Flow cytometry
☒	☐	MRI-based neuroimaging

Animals and other organisms

Policy information about studies involving animals; ARRIVE guidelines recommended for reporting animal research

Laboratory animals	Experiments were performed on 27 adult transgenic mice of either sex (4 – 10 months) expressing the Cre recombinase in specific subsets of interneuron on a C57BL/6J background.
Wild animals	This study did not involve wild animals.
Field-collected samples	This study did not involve field-collected samples.
Ethics oversight	All experimental procedures were conducted according to the UK Animals Scientific Procedures Act (1986). Experiments were performed at University of Sussex under personal and project licenses granted by the Home Office following review by the Animal Welfare and Ethics Review Board.

Note that full information on the approval of the study protocol must also be provided in the manuscript.
